# EBF1 drives hallmark B cell gene expression by enabling the interaction of PAX5 with the MLL H3K4 methyltransferase complex

**DOI:** 10.1038/s41598-021-81000-5

**Published:** 2021-01-15

**Authors:** Charles E. Bullerwell, Philippe Pierre Robichaud, Pierre M. L. Deprez, Andrew P. Joy, Gabriel Wajnberg, Darwin D’Souza, Simi Chacko, Sébastien Fournier, Nicolas Crapoulet, David A. Barnett, Stephen M. Lewis, Rodney J. Ouellette

**Affiliations:** 1 Atlantic Cancer Research Institute, Pavillon Hôtel-Dieu, 35 Providence Street, Moncton, NB E1C 8X3 Canada; 2 Department of Chemistry and Biochemistry, Université de Moncton, Moncton, New Brunswick Canada; 3 Present Address: The Centre for Applied Genomics, Toronto, ON Canada

**Keywords:** Cancer, Molecular biology, Diseases, Oncology

## Abstract

PAX5 and EBF1 work synergistically to regulate genes that are involved in B lymphocyte differentiation. We used the KIS-1 diffuse large B cell lymphoma cell line, which is reported to have elevated levels of PAX5 expression, to investigate the mechanism of EBF1- and PAX5-regulated gene expression. We demonstrate the lack of expression of hallmark B cell genes, including *CD19*, *CD79b*, and *EBF1*, in the KIS-1 cell line. Upon restoration of EBF1 expression we observed activation of *CD19*, *CD79b* and other genes with critical roles in B cell differentiation. Mass spectrometry analyses of proteins co-immunoprecipitated with PAX5 in KIS-1 identified components of the MLL H3K4 methylation complex, which drives histone modifications associated with transcription activation. Immunoblotting showed a stronger association of this complex with PAX5 in the presence of EBF1. Silencing of KMT2A, the catalytic component of MLL, repressed the ability of exogenous EBF1 to activate transcription of both *CD19* and *CD79b* in KIS-1 cells. We also find association of PAX5 with the MLL complex and decreased CD19 expression following silencing of KMT2A in other human B cell lines. These data support an important role for the MLL complex in PAX5-mediated transcription regulation.

## Introduction

PAX5 is an essential transcription factor in B lymphocyte differentiation that plays a role in the activation of B cell hallmark genes such as *CD19*, which encodes a transmembrane protein involved in B cell signaling and antigen response^1^. PAX5 is first expressed at the pro-B cell stage and its expression is maintained through subsequent B cell stages until it is downregulated during the transition into plasma cells^2^. PAX5 controls the switch from activated B cells into plasmablasts in part by repressing the expression of the transcription factors PRDM1 and XBP1^3^. PAX5 also serves to repress differentiation to other hematopoietic cell types^4^; for example, it represses NOTCH1 expression and thereby impairs T cell development^5^. PAX5 contributes to the transcriptional activation of B-cell-hallmark genes such as *CD19* and *CD79b* by interacting with other proteins. These PAX5 interacting proteins include components of the basal transcriptional apparatus such as RNA polymerase II, the TATA binding protein (TBP) and TBP-associated factors (TAFs)^6^, as well as proteins involved in chromatin remodeling and histone modification^7^.

The KIS-1 cell line originated from a patient with Ki-1-positive (Indicating the presence of TNFRSF8, also known as CD30) diffuse large B cell lymphoma (DLBCL)^8^. It was described as a DLBCL based on positive staining for HLA-DR and CD45 and negative staining for CD20 and antigens specific to other cell types. Class switch recombination of the JH locus (encoding a segment of the immunoglobulin heavy chain, IgH) and expression of lambda light chain suggest that the KIS-1 cell line originated from an activated B lymphocyte undergoing plasma cell differentiation. The KIS-1 cell line has a t(9;14)(p13;q32) translocation that brings the *PAX5* coding region and its promoter into the vicinity of the strong Eµ enhancer of the IgH gene^9–11^, which is highly active in immunoglobulin-secreting plasma cells. Consistent with this, KIS-1 DLBCL cells have very strong expression of PAX5^11–13^ at a time in B cell differentiation when PAX5 is usually switched off. Nevertheless, despite high PAX5 expression, Hamada et al.^12^ demonstrated an absence of *CD19* mRNA in KIS-1 cells, suggesting that PAX5 is not sufficient to drive expression of this hallmark B cell gene. We reasoned that restoration of expression of a missing protein partner of PAX5 might restore CD19 expression in KIS-1 DLBCL cells.

We here report an expanded characterization of gene expression in KIS-1 cells, confirm the lack of CD19 expression and demonstrate reduced expression of other B cell hallmark genes including *CD79b* and *EBF1*. Exogenous expression of the transcription factor EBF1, a transcription factor required for the expression of certain PAX5-regulated genes^14^, is sufficient to restore expression of *CD19*, *CD79b* and other B cell-specific genes to KIS-1 DLBCL cells. We further demonstrate that this transcriptional activation is mediated in part by increased association of PAX5 with the MLL (mixed-lineage leukemia) H3K4 methyltransferase complex, including the catalytic component KMT2A, in the presence of EBF1. Our results also support a role for the MLL complex, in association with PAX5 and EBF1, for B cell-specific transcription regulation in other human B cell lines.

## Results

### KIS-1 cells lack hallmark B cell gene expression

**Figure 1:**
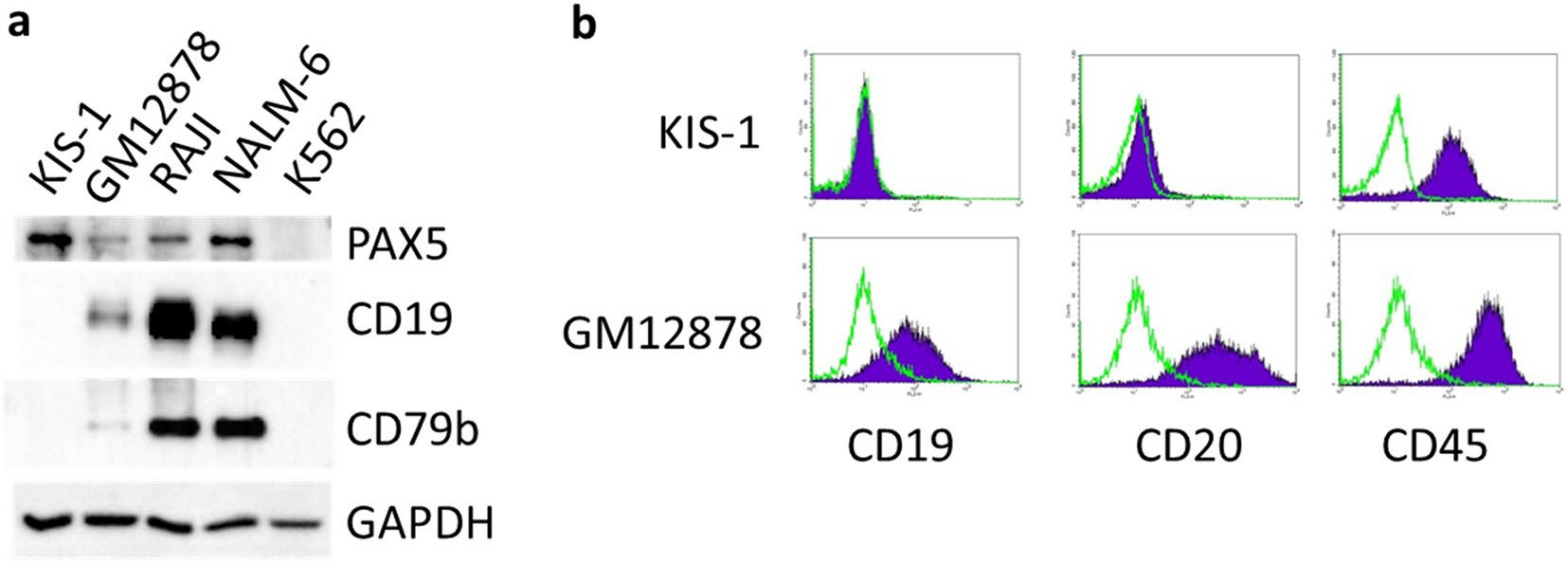
Expression of PAX5 and other B cell hallmarks in KIS-1. **a** Western blotting to show the expression of PAX5, CD19 and CD79b in KIS-1 whole cell extracts in comparison to three B cell lines (GM12878, RAJI and NALM-6) and a non-B lymphocyte line (K562). GAPDH is included to demonstrate equal loading. **b** Expression of CD19, CD20 and CD45 in KIS-1 versus GM12878 cells shown by flow cytometry. Antibody-labelled cells are indicated in purple, unlabeled cells are indicated in green. CD45 is a commonly expressed leukocyte antigen and is included as a positive control.

The KIS-1 DLBCL cell line was previously reported to have high expression of *PAX5* mRNA and PAX5 protein^11–13^ and undetectable expression of *CD19* mRNA^12^. We used Western blot analyses to compare the protein expression level of PAX5, CD19 and CD79b (also PAX5-regulated), in KIS-1 cells in addition to several other B cell lines (Fig. 1a). PAX5, CD19 and CD79b are all absent in K562 (a non-B Chronic Myelogenous Leukemia cell line). PAX5 is expressed more strongly in KIS-1 cells than in the other B cell lines investigated. By contrast, CD19 and CD79b are absent in KIS-1 cells but are expressed in GM12878 (an Epstein-Barr Virus-transformed B lymphocyte), RAJI (Burkitt lymphoma) and Nalm-6 (Acute Lymphoblastic Leukemia) cells. We further demonstrate the lack of CD19 and CD20 (another B-lymphocyte-specific cell surface protein) expression in KIS-1 cells by flow cytometry (Fig. 1b). These results confirm and extend previous findings and characterize KIS-1 as having lost B cell specific gene expression despite strong expression of PAX5.

**Figure 2:**
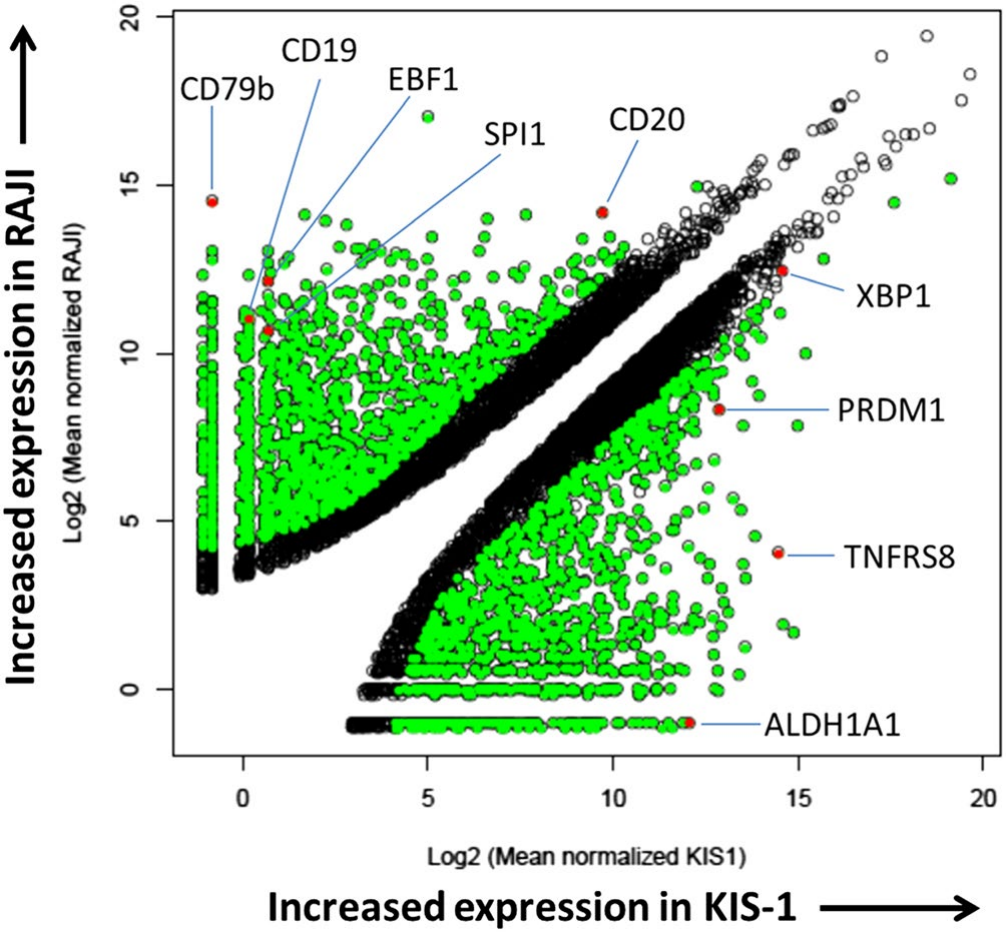
Differential gene expression in KIS-1 versus RAJI cells as determined by RNA-seq. Genes with significantly different gene expression levels between the two cell lines are indicated in green. Genes discussed in the text are indicated in red. Volcano plot generated with ggplot2 (version 3.0.0) R library (see “Methods”).

To further characterize gene expression in KIS-1 DLBCL cells we used next-generation sequencing, specifically RNA-seq, to compare this cell line to the RAJI cell line, which has much lower *PAX5* expression yet strong expression of both CD19 and CD79b (Fig. 1a). 2913 genes are differentially expressed (with a log^2^ fold change of ≥ 2.5 (5.7x) and False Discovery Rate (FDR) value of < 0.05) between the two lines: 1557 genes are downregulated and 1356 genes are upregulated in KIS-1 cells relative to RAJI cells (Supplementary Table 1). Results of genes most differentially expressed in each cell line is shown in Table 1. *PAX5* is expressed 2.4 × more strongly in KIS-1, consistent with the Western blot results (Fig. 1a). The top ten genes down-regulated in KIS-1 cells according to this analysis include heavy- and light-chain immunoglobulin genes and *CD79b*. *CD19* and *CD20* are also strongly and significantly down-regulated (fold changes of 2815 × and 22x, respectively) in KIS-1 cells, as are *SPi1* and *EBF1* (1015 × and 1932x, respectively). SPi1 and EBF1 are transcription factors essential for B cell differentiation whose expression is normally terminated during the plasmablast transition. Figure 2 summarizes the differentially-expressed genes between these two cell lines. 1317 of the 1557 down-regulated genes in KIS-1 were mapped using DAVID 2.0^15^ to five KEGG pathways with a fold enrichment of at least 2.5: Primary immunodeficiency (hsa05340), Osteoclast differentiation (hsa04380), B cell receptor signaling pathway (hsa04662), Arginine and proline metabolism (hsa00330), and NF-κB signaling pathway (hsa04064). The B cell receptor signalling pathway is represented by 10 genes: *CD19*, *CD79b*, *SYK*, *CD72*, *BTK*, *NFATC1*, *VAV1*, *FCGR2B*, *FOS/AP-1* and *BCAP*. These data clearly demonstrate that KIS-1 DLBCL cells lack gene expression characteristic of PAX5-expressing B cells like RAJI, despite having an elevated level of PAX5 expression.

**Table 1:** List of most significantly up-regulated and down-regulated genes in KIS-1 versus RAJI cells as determined by RNA-seq.

	KIS1 mean	RAJI mean	log^2^ Fold Change	*p* value	*p*_adj_
IGHM	0	166,655.1998	− 19.7346	3.78E−37	1.94E−34
IGHV3-21	0	19,103.9253	–16.6097	1.04E−26	1.51E−24
ST14	0	10,358.3341	–15.7266	2.96E−24	3.43E−22
CD79B	0.5649	23,122.6324	**–**15.4408	1.20E−24	1.44E−22
TCL1A	0	6160.0247	−14.9768	3.72E−22	3.60E−20
IGHV1-69	0	3511.1722	−14.1658	4.98E−20	3.80E−18
IDH2	0	3159.8325	−14.0138	1.48E−19	1.06E−17
SERPINA9	0.5649	8432.8389	− 13.9856	1.68E−20	1.35E−18
IGKV3-20	0	3035.7311	− 13.9559	1.76E−19	1.25E−17
IGHG1	0	2997.9458	− 13.9379	1.40E−19	1.02E−17
SEMA4C	1468.6114	0	13.0477	2.95E−17	1.61E−15
ALDH1A1	4257.3685	0.5	13.1376	2.70E−17	1.48E−15
ATP9A	30,003.313	3.2228	13.1626	1.62E−67	1.25E−63
BLVRA	1651.8437	0	13.2173	1.02E−17	6.08E−16
ARNTL2	1911.8631	0	13.428	1.31E−17	7.69E−16
MSC	2551.8359	0	13.8449	1.37E−18	8.79E−17
TMEM173	2734.9963	0	13.9447	1.49E−19	1.07E−17
EOMES	3040.8551	0	14.0977	7.49E−20	5.60E−18
LHX2	3201.0322	0	14.1717	3.33E−20	2.58E−18
ANKRD30A	5323.245	0	14.9054	1.01E−20	8.40E−19

Genes including mean RNA counts, log^2^ fold change and *p* value.

### *PAX5* is wildtype in KIS-1 cells and *CD19* and *CD79b* genes can be activated by treatment with a DNA demethylation agent

To test the possibility that the lack of *CD19* and *CD79b* expression in KIS-1 cells is due to inactivating mutations within the *PAX5* gene, we analyzed the KIS-1 RNA-seq data for insertion/deletions (indels) and single nucleotide polymorphisms (SNPs). Although indels and SNPs were identified in the 3ʹ and 5ʹ UTRs and intronic regions of the *PAX5* gene (Supplementary Table 2), none were identified in the coding region. These data, along with the PAX5 protein migrating at the expected molecular weight as determined by Western blot analysis, together support the idea that the translocated *PAX5* gene has a wildtype sequence and is therefore potentially functional.

**Figure 3:**
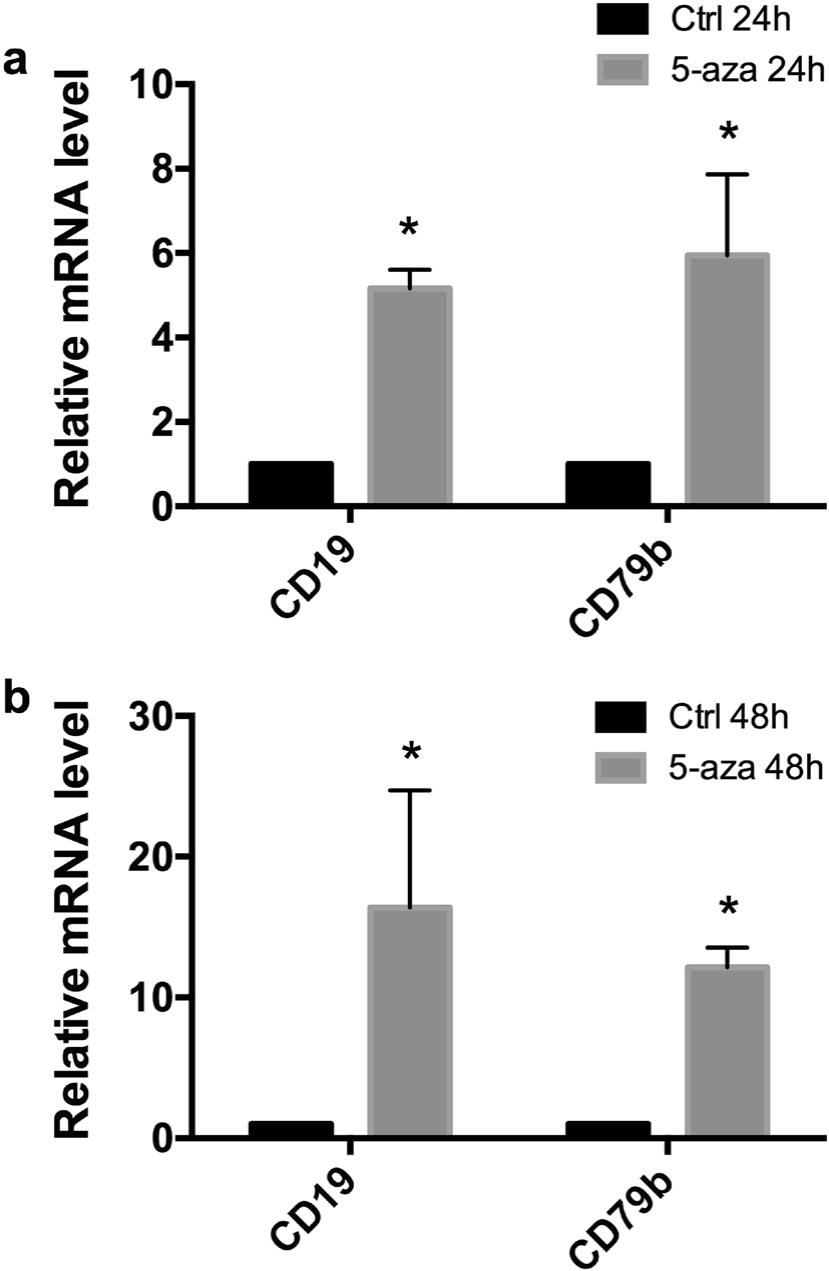
Activation of *CD19* and *CD79b* expression in KIS-1 following treatment with 5-azacytidine (5-azaC) for 24–48 h shown by RT-qPCR. Standard error of the mean is shown. Data represents 3 independent experiments. Statistical analysis was performed by multiple *t* test of log transformed fold change. Statistically-significant comparisons (*p* < 0.05) are indicated by *.

The failure to detect CD19 and CD79b expression in KIS-1 cells could also arise due to decreased transcription mediated by DNA methylation of regulatory regions of the *CD19* and *CD79b* genes or of genes that regulate *CD19* and *CD79b*. We therefore treated KIS-1 cells with 5-azacytidine, a compound that reduces DNA methylation at CpG sequences and can thereby drive transcriptional activation^16^. This treatment was able to increase the abundance of both CD19 and CD79b mRNAs (Fig. 3), suggesting that epigenetic silencing does indeed play a role in the lack of expression of CD19 and CD79b in KIS-1 DLBCL cells.

### PAX5 regulation of gene expression in KIS-1 cells

**Figure 4:**
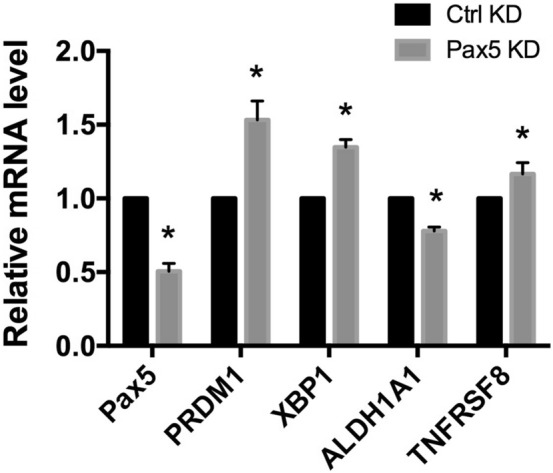
Gene expression changes in KIS-1 following siRNA-mediated silencing of *PAX5* for 48 h shown by RT-qPCR. Standard error of the mean is shown. Data represents 3 independent experiments. Statistical analysis was performed by multiple t-test. Statistically-significant comparisons (*p* < 0.05) are indicated by *.

PRDM1 (also known as BLIMP-1) and XBP1 are transcription factors whose expression increases near the plasmablast transition. Consistent with the assignment of KIS-1 as an activated B lymphocyte undergoing plasma cell differentiation, RNA-seq (see Supplementary Table 1) indicates that both *PRDM1* and *XBP1* are upregulated in KIS-1 cells (23× and 4×, respectively) relative to RAJI, a cell line derived from B cells at an earlier stage of differentiation. PAX5 is known to repress PRDM1 and XBP1 expression, and the interplay of these transcription factors is critical to proper B cell development ^3,17^. In order to explore the idea that temporally-inappropriate PAX5 overexpression is limiting further differentiation in KIS-1 through regulation of PRDM1 and XBP1, we used siRNA to silence PAX5 expression and found that reduced PAX5 expression does indeed drive an increase in PRDM1 expression, as well as a statistically-significant increase in XBP1 expression (Fig. 4). While these results suggest that high PAX5 expression is limiting the levels of these transcription factors in KIS-1 cells, it is clearly not sufficient to fully repress their expression. The regulation of the expression of these genes must involve additional factors.

We also explored whether high PAX5 expression in KIS-1 cells is involved in the strong upregulation of two genes, *ALDH1A1* (1912x) and *TNFRSF8* (1380x), relative to their expression in RAJI cells (based on our RNA-seq data). ALDH1A1 is a marker for cancer stem cells, can be a therapeutic target in cancer, and is involved in cyclophosphamide resistance^18^. It is therefore of interest to understand the regulation of its gene expression. Silencing of PAX5 significantly decreased expression of ALDH1A1, suggesting that PAX5 does indeed play a role in its expression (Fig. 4). TNFRSF8 (the Ki-1 antigen that gives KIS-1 its name; also known as CD30) is a target for cancer therapeutics and is a marker for Hodgkin’s lymphoma, anaplastic large-cell lymphoma, and germ cell tumours^19^, all of which are cancer cells that do not generally express significant levels of PAX5. PAX5 silencing did not strongly influence expression of TNFRSF8 (Fig. 4), indicating a minor PAX5 involvement in the regulation of the expression of TNFRSF8 in the KIS-1 cell line. Taken together, these results suggest that PAX5 participates in the regulation of gene expression in KIS-1 cells, despite being unable to activate transcription of either *CD19* or *CD79b*.

### Exogenous expression of EBF1 restores CD19 and other B cell hallmark gene expression in KIS-1 cells

**Figure 5:**
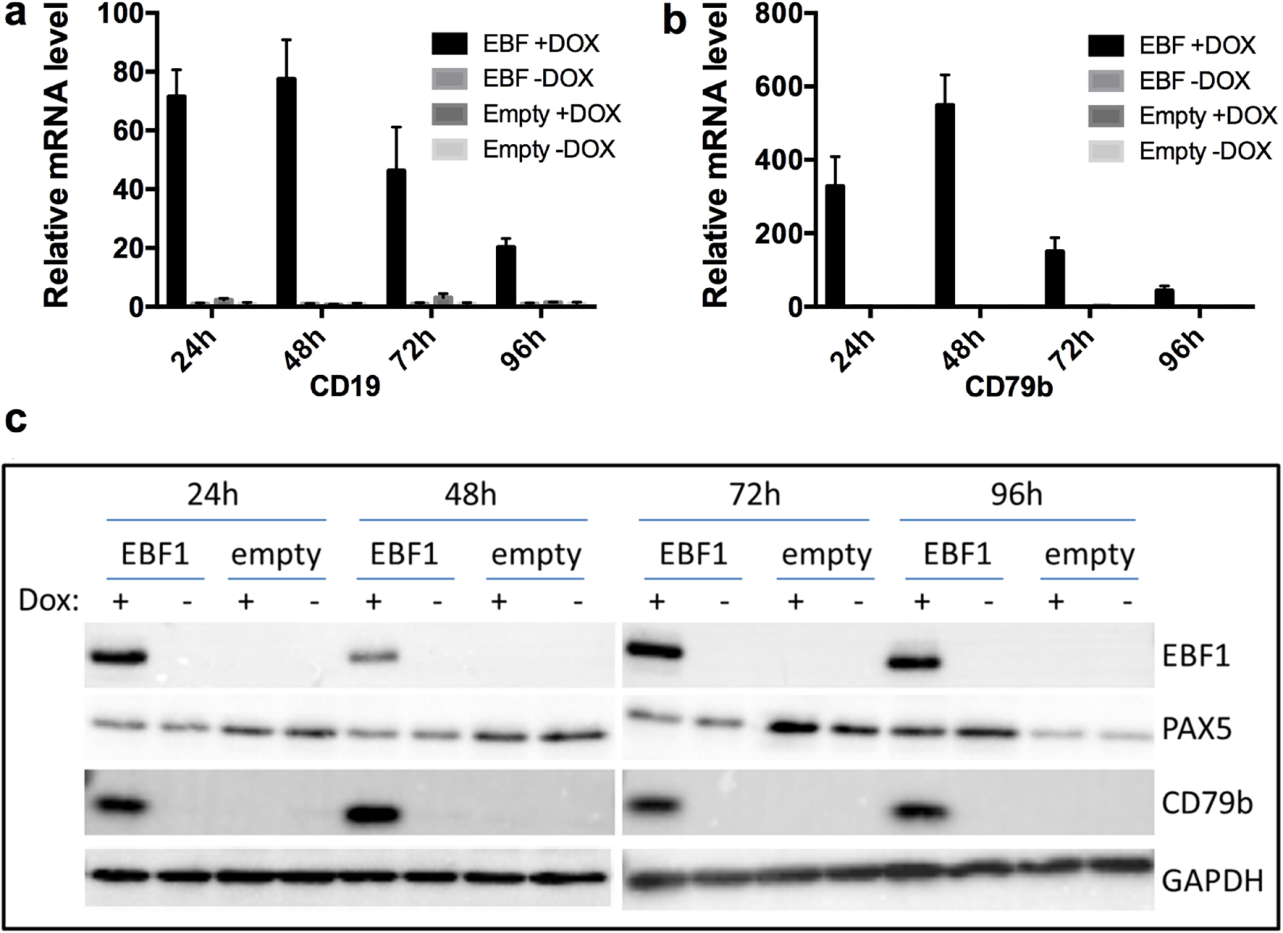
Activation of CD19 and CD79b expression in KIS-1 following doxycycline-induced expression of EBF1 for 24–96 h. Activation of *CD19* (**a**) and *CD79b* (**b**) shown by RT-qPCR. Activation of CD79b expression shown by Western blotting (**c**). Data represents 3 independent experiments.

EBF1 is a critical transcription factor in early B cell development and is necessary for the expression of certain PAX5-regulated genes, including *CD19*^14^. We hypothesized that the absence of EBF1 expression in KIS-1 cells, as determined by our RNA-seq analysis, might therefore explain the lack of CD19 expression. KIS-1 cells were stably transduced with a lentiviral vector that encodes a doxycycline (DOX)-inducible *EBF1* gene, thereby creating a cell line referred to here as ‘KIS-1 + EBF1’. EBF1 expression was subsequently induced for 24–96 h by addition of DOX. CD19 and CD79b mRNA expression was found to be strongly activated by the induction of EBF1 expression and CD79b protein expression was also restored (Fig. 5). CD19 protein remained undetectable by Western blot over the course of the experiment (data not shown). DOX induction of cells stably transduced with an empty vector (‘KIS-1 + empty’) served as a negative control.

To extend these results, we used RNA-seq to obtain a global view of EBF1-driven gene expression changes after 48 h of DOX induction. As a control for the effects of DOX, KIS-1 + empty cells were treated with DOX or left untreated; comparison of these gene expression profiles showed no differentially-regulated genes (Supplementary Table 3A), demonstrating that DOX itself does not significantly affect gene expression in our experiment. KIS-1 + EBF1 without DOX compared to KIS-1 + empty without DOX induction did show a significant (18x) increase of EBF1 expression, likely due to leaky expression of EBF1 in KIS-1 + EBF1 cells in the absence of DOX (Supplementary Table 3B). Nevertheless, this undesired expression of EBF1 mRNA did not result in detectable EBF1 protein expression (see Fig. 5b, c) and caused no additional gene expression changes, thus it did not interfere with downstream analyses of the effects of DOX-induced EBF1 expression.

Comparison of KIS-1 + EBF1 cells with or without DOX treatment revealed 167 upregulated genes and 0 downregulated genes (Supplementary Table 3C). Comparison of KIS-1 + EBF1 cells treated with DOX to KIS-1 + Empty cells treated with DOX showed 160 upregulated genes and 1 downregulated gene (a 7 × reduction in *KLF15*) (Supplementary Table 3D). Combining these two datasets revealed 138 shared upregulated genes, including both *CD79b* and *CD19*, and no shared downregulated genes following expression of EBF1 in KIS-1 cells (Supplementary Table 3E). 121 of 138 shared genes were assigned a category using the Panther Overrepresentation test (FDR < 0.05)^20^. The ‘Biological Processes’ category shows enrichment of two GO terms: GO:1903039 (positive regulation of leukocyte cell–cell adhesion; FES (fold enrichment score) = 5.76; 8 genes) and GO:0045321 (leukocyte activation; FES = 2.94; 16 genes). Interestingly, this analysis also highlighted major changes in the expression of genes coding for cell surface markers resulting from re-establishment of EBF1 expression in KIS-1 DLBCL cells: in the ‘Cellular Component category’, GO:0098797 (plasma membrane protein complex) had the highest fold enrichment score (FES; 5.92) and included 9 genes, while GO:0005887 (Integral component of the plasma membrane) had an FES of 3.06 and included 31 genes. These data indicate that EBF1 predominantly activates rather than represses transcription in the KIS-1 cell line under the conditions tested, and that the activated genes include B cell hallmark genes.

### Proteomic analysis of PAX5-interacting proteins in KIS-1 cells

**Figure 6:**
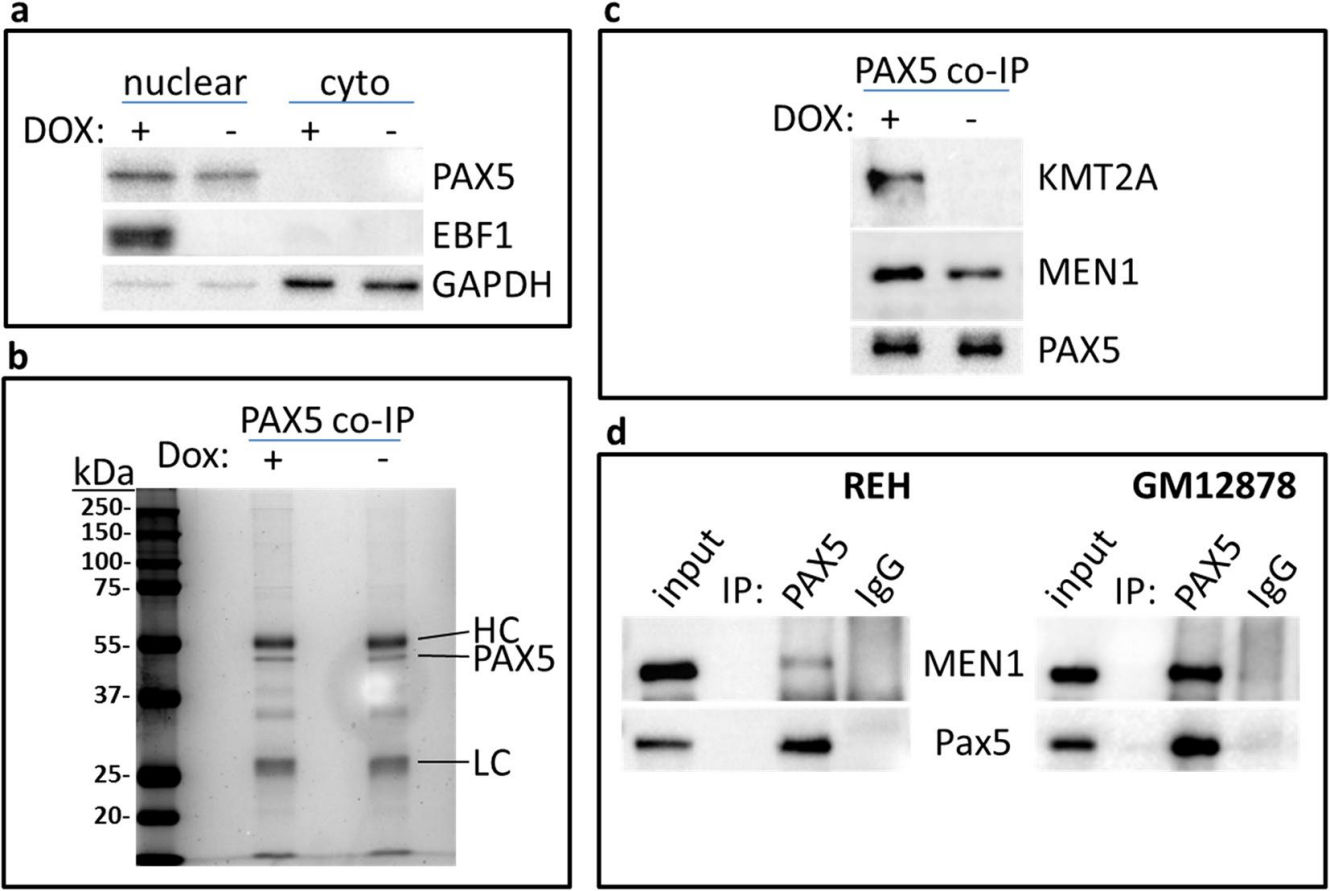
Confirmation of PAX5 interacting proteins in KIS-1 and REH. (**a**) Western Blot to show EBF1 and PAX5 expression in KIS-1 + EBF1 nuclear and cytoplasmic (cyto) extracts with and without DOX-induction for 48 h. (**b**) Coomassie Blue-staining of PAX5 co-immunoprecipitated material from KIS-1 + EBF1 nuclear extracts with or without doxycycline induction for 48 h. (**c**) Western blot to show KMT2A co-immunoprecipitation with PAX5 from KIS-1 + EBF1 nuclear extracts with and without doxycycline induction for 48 h. (**d**) Western blot to show MEN1 co-immunoprecipitation with PAX5 from REH and GM12878 nuclear extracts.

To further investigate the mechanisms that underlie EBF1-mediated activation of gene expression in KIS-1 cells, we sought to identify proteins that interact with PAX5 in the presence or absence of EBF1. KIS-1 + EBF1 cells were treated with DOX or left untreated and nuclear protein extracts were collected and subjected to co-immunoprecipitation (co-IP) using either an anti-PAX5 antibody or non-specific control antibodies. Western blot analysis confirmed the induction of EBF1 expression following DOX treatment and a similar level of PAX5 expression under these conditions (Fig. 6a). Following immunoprecipitation of PAX5, the Coomassie Blue staining pattern of associated proteins was very similar for both DOX treated and untreated KIS-1 + EBF1 cells, including a prominent PAX5 band (Fig. 6b).

Mass spectrometry was used to identify proteins that co-precipitate with PAX5 in either the presence or absence of EBF1, with a requirement for at least two unique peptides per protein identified in each of two replicate samples. PAX5 was identified in each replicate by at least 16 unique peptides. 77 and 66 proteins were identified in PAX5 co-IPs from DOX treated and untreated samples, respectively, to give a total of 93 PAX5-interacting proteins in KIS-1 cells (Supplementary Table 4). As expected, proteins involved in transcription are well represented: 31 of 93 (33%) proteins were assigned to the Panther ‘Biological Process’ category GO:0006351 (transcription, DNA-templated; 2.81 × enriched), including 6 TAFs (TATA-box binding protein associated factors). Histone modification and chromatin remodeling complexes are also present (‘Cellular Component’ category), including the 8 members of the MLL H3K4 methylation complex (GO:0071339, 65.22 × enriched). One enriched ‘Biological Process’ category of note is GO:0000398 (mRNA splicing, via spliceosome, 14.1x, 17 proteins). These data support a role for PAX5 in complexes involved in transcription regulation and indicate a previously unappreciated association of PAX5 with splicing complexes. To our knowledge, this is the first global analysis of PAX5 interacting proteins in a human cell line.

We then sought to identify proteins with differential binding to PAX5 in the presence versus the absence of EBF1 to identify proteins and protein complexes that might explain how EBF1 is able to activate transcription of PAX5-regulated genes. 21 proteins were identified with minor (1.5x) increases in binding to PAX5 in the presence of EBF1, based on the number of unique peptides in each replicate (Supplementary Table 4), whereas 4 proteins showed decreased binding to PAX5 in the presence of EBF1 expression. The group of proteins that interact slightly more strongly with PAX5 in the presence of EBF1 includes the MLL complex members (GO:0071339) RBBP5, TAF6 and the catalytic component of MLL, KMT2A (originally named MLL). The MLL complex drives tri-methylation of H3K4 at gene promoters, a histone modification strongly correlated with transcription activation. This complex is therefore an excellent candidate for mediating transcriptional activation in concert with PAX5. None of the 21 proteins with increased PAX5 association had significantly increased expression of their respective mRNAs in the presence of EBF1, as determined by RNA-seq (see Supplementary Tables 3C and 3D), suggesting that their increased presence in PAX5 immunoprecipitates from EBF1-expressing cells is not due to a simple increase in abundance. The lack of increased expression in the presence of EBF1 was further confirmed for KMT2A and its interactor MEN1 by immunoblotting (Supplementary Figure S1).

### EBF1 enables the interaction of the MLL complex with PAX5

Western blot analysis confirmed that the interaction of PAX5 with KMT2A is strongly enhanced in the presence of EBF1 (Fig. 6c). Further, the interaction of PAX5 with MEN1, a protein unique to complexes with either a KMT2A or KMT2B catalytic subunit, was also enhanced in the presence of EBF1 (Fig. 6c). These results suggest that EBF1 may activate transcription by enabling the interaction of PAX5 with KMT2A as part of the MLL complex. Closer examination of the proteomics data from PAX5 co-immunoprecipitations revealed at least one peptide identifying the four subunits common to all KMT2 complexes (ASH2L, RBBP5, WDR5 and DPY30) (see Rao and Dou, 2015 for subunit composition of KMT2 complexes) (Supplementary Table 5) as well as the two subunits unique to KMT2A/B complexes (KMT2A and MEN1). By contrast, only two proteins (KMT2D and NCOA6) unique to another KMT2 complex (KMT2C/D, also named MLL3/4) were identified by mass spectrometry, and these by only one peptide each. This suggests that PAX5 is predominantly associated with KMT2 complexes where KMT2A is the catalytic component.

To further confirm the PAX5-MLL interaction in B cells other than KIS-1, we demonstrated by co-immunoprecipitation and immunoblotting an interaction between PAX5 and the MLL complex component MEN1 in the REH and GM12878 cell lines (Fig. 6d). Furthermore, mass spectrometry of interacting proteins of PAX5 in the B cell lines NALM6 and RAJI identified KMT2A by multiple unique peptides in at least one of the two replicates for both cell lines, as well as RBBP5, WDR5 and HCFC1 (Supplementary Table 5). Interaction of ASH2L and MEN1 with PAX5 was also identified in RAJI cells. Together, these data strongly support the interaction of the MLL complex (with KMT2A as the catalytic component) with PAX5 in human B cells.

### A role for KMT2A in EBF1- and PAX5-regulated transcription

**Figure 7:**
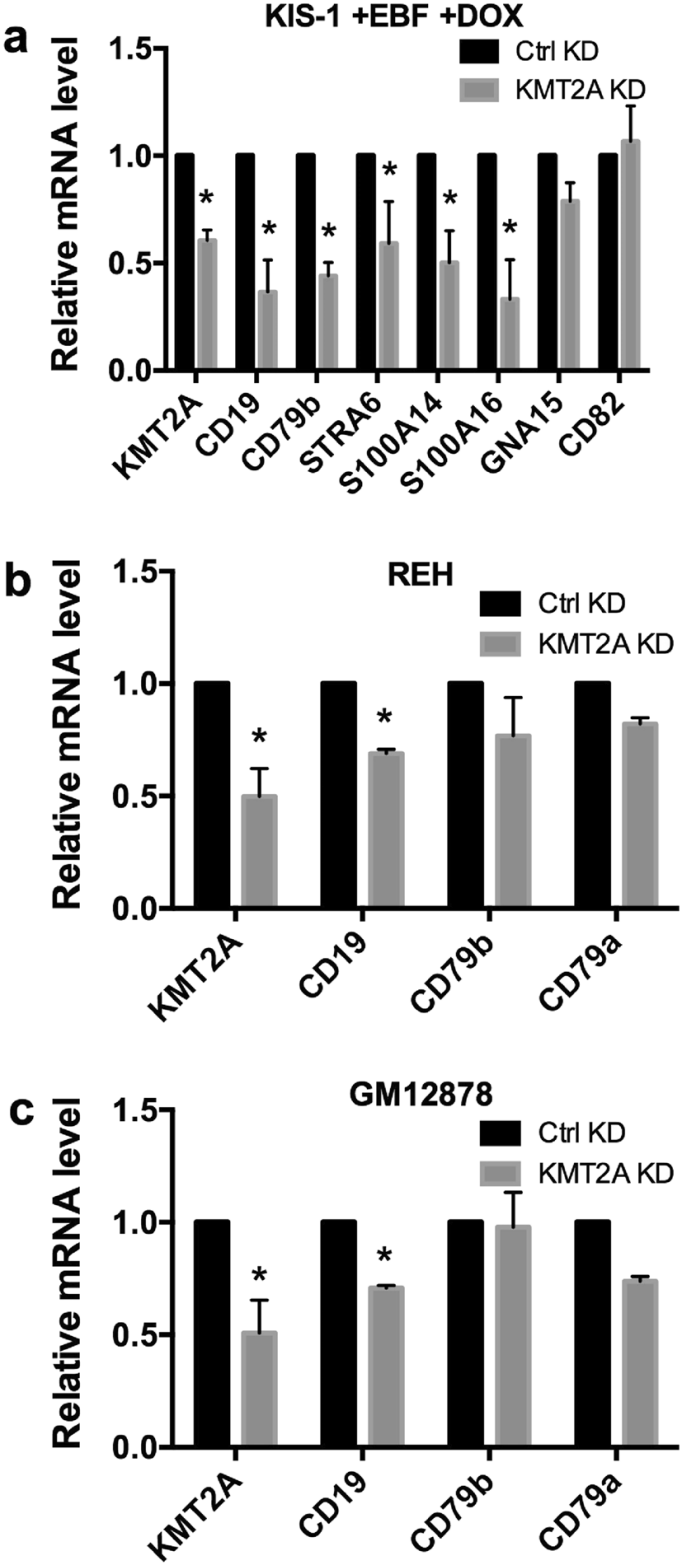
Gene expression changes following siRNA-mediated silencing of KMT2A shown by RT-qPCR. In KIS-1 + EBF1 induced with DOX for 24 h (**a**), REH (**b**) and GM12878 cells (**c**). Standard error of the mean is shown. Data represents 3 independent experiments for KIS-1 + EBF1 and GM12878 and 4 experiments for REH.Statistical analysis was performed by multiple *t* test. Statistically significant comparisons (*p* < 0.05) are indicated by *.

In order to explore whether KMT2A is necessary for EBF1-driven activation of transcription in KIS-1 + EBF1 cells, we silenced KMT2A using siRNA and then induced EBF1 expression. We found that EBF1-driven activation of both *CD19* and *CD79b* was significantly reduced when levels of KMT2A were reduced in KIS-1 cells (Fig. 7a). We further observed decreases (though in some cases not reaching statistical significance) in the levels of additional EBF1-activated genes previously identified by RNA-seq: *STRA6*, *S100A14*, *S100A16* and *GNA15*. By contrast, the level of another highly upregulated gene following EBF1 expression, *CD82*, was unchanged by KMT2A silencing.

To test whether involvement of KMT2A in EBF1-/PAX5-regulated transcription is specific to the KIS-1 cell line or a general B cell phenomenon, we next silenced KMT2A in the REH (Fig. 7b) and GM12878 (Fig. 7c) cell lines and found that the expression of CD19 was significantly reduced in both while expression of CD79a and CD79b were decreased but not statistically significant. Taken together, these data support activation and maintenance roles for KMT2A and the MLL complex in EBF1- and PAX5-regulated B cell hallmark gene expression, including in the expression of CD19. ChIP analysis (Supplementary Figure S4) demonstrates that EBF1 expression surprisingly does not lead to an increase of di- and tri-methylation of H3K4 (the principal substrate of KMT2A) at cd19 and cd79B promoters, where EBF1 and PAX5 are known to bind. This suggests that the increased interaction of the KMT2A complex with PAX5 does not drive cd19 and cd79B transcription simply by increasing H3K4 methylation.

## Discussion

To accomplish its crucial roles in B cell development, the transcription factor PAX5 relies on protein partners such as mi-2/NuRD and SWI-SNF^7,21,22^, ETS1, p300^23^, CREBBP, DAXX^24^ and TLE4^25–27^ to regulate transcriptional activation and repression. For transcription activation, the roles of PAX5 partner proteins include rendering chromatin accessible for PAX5 binding as well as manipulating histone post-translational modifications. EBF1 has been reported to physically interact with PAX5^28^, and it works together with PAX5 to regulate the transcription of many genes during B cell development^29^. In the present study we show that EBF1 restores expression of *CD19*, *CD79b* and other B-cell-specific genes to the PAX5-expressing KIS-1 cell line.

Expression of EBF1 has been previously reported to stimulate B-cell-specific gene expression in cells that normally lack EBF1, and it can accomplish this even in non-B lymphocytes: for example, Akerblad et al.^30^ showed activation of *CD79b* following EBF1 expression in HeLa cells; Bohle et al.^31^ demonstrated EBF1-driven activation of *CD19*, *CD79a* and *CD79b* in Hodgkin Lymphoma cell lines; Gao et al.^22^ demonstrated *CD19* and *CD79a* expression in a murine plasmacytoma system that required exogenous expression of both EBF1 and PAX5; and Li et al.^14^ introduced EBF1 expression into pre-pro-B murine cells lacking EBF1 and demonstrated activation of both *PAX5* and *CD19* expression. Finally, we have observed that exogenous EBF1 expression in HEK293T cells drives *CD19* and *CD79b* mRNA expression (Supplementary Figure S2). EBF1 can thus generally override a cell’s programming to express B-cell hallmark genes.

Indeed, EBF1 is known as a ‘pioneering’ transcription factor that prepares chromatin regions for binding of additional nuclear factors, including PAX5, and subsequent transcription activation. An elegant recent demonstration of this idea shows that EBF1 binds to its DNA recognition sequences even before the formation of accessible chromatin or CpG demethylation^14^. It will be of interest for future work to explore whether PAX5 can in fact bind at EBF1-activated genes such as *CD19* in KIS-1 cells prior to exogenous EBF1 expression. If PAX5 cannot bind its DNA recognition sequences, this may be related to decreased promoter accessibility or to increased CpG methylation in the absence of EBF1.

EBF1 plays roles beyond preparing chromatin for PAX5 binding in B cell gene expression; EBF1 is co-expressed with PAX5 throughout B cell development until the transition to plasma cells, and silencing of EBF1 (as well as PAX5) in the human B cell line REH results in loss of B cell-specific gene expression (Supplementary Figure S3), indicating that EBF1 is involved in maintaining gene expression as well as in its initial activation. Here we broaden the scope of known protein interactors of PAX5 and demonstrate that one of the roles of EBF1 is to modulate protein interactors of PAX5 that drive and maintain transcription of PAX5-regulated genes. Two interactors of PAX5 influenced by EBF1 expression in KIS-1 cells are MEN1 and KMT2A, components of the MLL H3K4 tri-methylation complex. The interaction of PAX5 with the N-terminal portion of KMT2A had previously been noted by Liu et al.^32^ using exogenously-expressed, tagged proteins in HEK293T cells.

Restoration of PAX5 expression in *PAX5*^*-/-*^ mouse B cells results in PAX5 binding at PAX5-regulated gene promoters, activation of gene expression and H3K4 tri-methylation at these promoters^7^. Our data suggest that PAX5 drives the activation and maintenance of expression of B cell-specific genes such as *CD19* in human B cells through physical interaction with components of the MLL complex including MEN1 and KMT2A. Despite our identification of an EBF1-dependent interaction between PAX5 and the catalytic subunit of the MLL H3K4 tri-methylation complex, namely KMT2A, we failed to observe any changes in methylation status of the PAX5-regulated genes *CD19* and *CD79b* in KIS-1 cells upon expression of EBF1 (Supplementary Figure S4). These results may indicate that the PAX5 interaction with the MLL tri-methylation complex does not lead to changes in histone methylation at PAX5-target genes or such changes may occur outside of the regions examined in our assays. Moreover, the PAX5-MLL complex interaction could also cause changes in the methylation status of specific co-factors (such as mi-2/NuRD and SWI-SNF, ETS1, p300, CREBBP, DAXX and TLE4), whose upregulated expression could lead to activation of PAX5-target genes. Future experiments to determine the consequences of the PAX5-MLL interaction for global histone methylation should help us understand the underlying mechanisms that lead to activation of PAX5-target genes upon its interaction with the MLL tri-methylation complex. Nonetheless, our work continues the dissection of the order of events that proceeds transcription activation by PAX5, and explains in part why the presence of PAX5 alone is not sufficient to drive expression of all its regulated targets in KIS-1 cells.

Other KMT2 complexes have previously been shown to be associated with PAX5. In mouse B cells, PAX5 interacts with RBBP5^7^, a common component of all KMT2 complexes^33^. PAXIP1 (also known as PTIP) has been shown to interact with both PAX5^7^ and the closely-related Pax2^34^ and mediates transcription factor interactions specifically with MLL3/4 complexes (containing KMT2C or 2D)^35,36^. MLL3/4 complexes are largely responsible for mono-methylation of H3K4 at enhancers^33^. These data taken together with our data therefore suggest that PAX5 influences transcriptional activation through physical interaction with the complexes that modify histones at both enhancers and promoters of target genes.

Based on our results, KIS-1 could prove to be an excellent model for further study of the mechanisms that underlie EBF1’s roles in PAX5-mediated transcription regulation. As it is a human cell line, it provides useful comparison and contrast with well-studied mouse model systems. Of interest in future work will be the roles of proteins found to associate with PAX5 in various B cells, with a particular focus on those shown here to be recruited to PAX5 following EBF1 expression. Some of these proteins, such as those involved in RNA splicing, will expand our understanding of the roles of PAX5 in gene expression and highlight new roles for this key B cell transcription factor.

## Methods

### Growth of human lymphocytes

GM12878 cells were obtained from the Coriell Institute (Cambden, N.J., United States). REH (ACC-22), Nalm-6 (ACC-128), RAJI (ACC-319) and K562 (ACC-10) cells were obtained from The Leibniz Institute DSMZ—German Collection of Microorganisms and Cell Cultures (Braunschweig, Germany). KIS-1 cells were kindly provided by Dr. Momoko Nishikori (Kyoto University, Japan). All suspension cells were cultured in RPMI 1640 media supplemented with 15% FBS, 1 mM sodium pyruvate, 2 mM L-glutamine and 1 × Glutamax. Cells were diluted 1/3–1/5 with media roughly every three days to maintain log-phase growth.

### Immunoblotting

Immunoblotting was performed with standard techniques using 0.45 μm PVDF membranes pre-wetted with methanol. Primary antibodies used: α-PAX5 (sc-1974 and sc-1975) from Santa Cruz Biotechnology; α-CD19 (ab134114), α-CD79b (ab134103), α-EBF1 (ab126135), α-KMT2A (ab32400) and α-MEN1 (ab92443) from ABCAM; α-GAPDH (2275-PC) from Trevigen. HRP-conjugated secondary antibodies were purchased from Santa Cruz Biotechnology. HRP activity was detected using ECL Prime Western Blotting Detection Reagent (GE Healthcare). Experiments involving immunoblotting were performed in biological duplicate or triplicate; representative immunoblots are shown.

### Flow cytometry

1 × 10^6^ cells were resuspended in 500 µl PBS and incubated at 4C for 30 min with the following antibodies from Becton Dickinson: α-CD20 (cat. # 347673), α-CD19 (#555412) and α-CD45 (#555482). Labelled cells were analyzed on a FACSCalibur flow cytometer (Becton Dickinson) using the BD FACStation for Mac OS X. Experiments involving flow cytometry were performed in biological duplicate; representative data are shown.

### Cloning, generation of virus and establishment of stable cell lines

The coding sequence of the *EBF1* gene was PCR amplified from plasmid MHS6278-202758239 (Dharmacon) using the primers 5ʹ-CCCTCGTAAAGAATTATGTTTGGGATTCAGGAAAGCATCCAACG-3ʹ and 5ʹ-GTGTATACGGGAATTTCACATAGGAGGAACAATCATGCCAGATATCG-3ʹ and CloneAmp HiFi PCR Premix (Clontech). The resulting amplicon was cloned into the EcoRI site of the pLVX-TetOne-Puro Vector (Clontech) using In-Fusion cloning (Clontech). Plasmid was isolated using the ZymoPURE II kit (Zymo). The insert sequence was confirmed by Sanger sequencing.

Lentivirus production and purification were performed as previously described^37^. Briefly, 3.8 ml OptiMEM was supplemented with 200ul of PLUS reagent (Life technologies), 20ug of lentiCRISPRv2 plasmid (Addgene #52961) containing the gRNA of interest, 10ug of pVSV (Addgene #8454) and 15ug of psPAX2 (Addgene #12260) and incubated for 5 min at room temperature (RT). 100ul of TransIT-X2 Dynamic Delivery System (Mirusbio) was diluted in 3.9 ml OptiMEM, added to the plasmid mixture and incubated at RT for an additional 15 min. A T175 flask of HEK293T cells at roughly 90% confluence was incubated for 1 h in 13 ml of OptiMEM and then the plasmid and transfection reagent mixture was added and incubated with the cells for 6 h at 37 °C. Media was then removed and cells were resuspended in DMEM High glucose media supplemented with 10% FBS and 1% BSA. After 60 h incubation at 37 °C, media was collected and centrifuged for 10 min at 3000 rpm at 4 °C to remove cell debris. The supernatant fraction was filtered using a 0.45 µm Acrodisc syringe filters (Pall laboratory), and viral particles were concentrated using Lenti-X Concentrator (Clontech) following the manufacturer’s protocol. The lentivirus concentration was titered using Lenti-X GoStix cassettes (Clontech).

Cells were transduced as previously described^38^ with modifications. Briefly, 7.5 × 10^5^ cells were resuspended in 1 ml media supplemented with 10ug/ml DEAE-Dextran sulfate (Sigma) and placed in one well of a 12 well plate. 20 ul of concentrated lentiviral particles was added per well. After 24 h, 1 ml of fresh media was added per well and cells were incubated for an additional 24 h. Cells were centrifuged at 700 g at RT for 10 min. Media containing free virus was discarded and the cell pellet was resuspended in fresh media supplemented with 3ug/ml puromycin. Puromycin selection was maintained for at least 7 days or until all uninfected control cells were killed by the antibiotic, as determined by trypan blue staining.

### Doxycycline treatment

KIS-1 cells stably-infected with lentiviral vectors were induced with 100 ng/ml doxycycline (DOX+) or vehicle (water; DOX-) for 24-96 h. Fresh doxycycline was added at 48 h post-induction.

### RNA-seq and data analysis

RNA was prepared by centrifugation of 5 × 10^6^ cells for 3 min at 700 g to remove media followed by lysis of cells in 1 mL Trizol (Invitrogen) and prepared according to the manufacturer’s protocol by adding 200 μL chloroform, mixing and then separating the phases by centrifugation. The aqueous phase was mixed 1:1 with 70% EtOH and loaded onto an RNeasy column (Qiagen). RNA purification was completed following the manufacturer’s protocol. The KIS-1 and RAJI RNA-seq datasets were based on two biological replicate samples. The KIS-1+EBF1+/−DOX and KIS-1+empty+/−DOX datasets were based on four biological replicate samples.

RNA quality was assessed using the Tape Station 2200 (Agilent) and RNA Screentape. Samples with an RNA integrity number of greater than 8 were selected for library preparation. Enrichment of Polyadenylated (poly(A)) RNA for whole transcriptome sequencing was performed using the Dynabeads mRNA DIRECT Micro Kit (Ambion). 100 ng of poly(A)-enriched RNA was used for library preparation using the Ion Total RNA-Seq Kit v2 (ThermoFisher). Library quality was assessed using the Tape Station 2200 and D1000 Screentape. Barcoded libraries were sequenced on the Ion Torrent Proton platform with two samples per chip using Ion PI chips.

Raw RNA-Seq data was processed using Cutadapt (version 1.14)^39^ to remove low-quality (with a Phred score of less than 15) and short (less than 30 nucleotides) reads. Mapping was performed using STAR (version 2.5.2)^40^ and then using Bowtie 2 (version 2.2.6)^41^ using the “local and very sensitive” option on the unmapped reads from STAR. Human genome version GRCh37/hg19 was used as the reference in all mapping steps, and SAMtools (version 1.6)^42^ was used to manipulate the data. We used Featurecounts (version 1.5.1)^43^ for read counting and DESeq 2 (version 1.18.1)^44^ to identify differentially expressed genes in the R statistical environment (version 2.4.4)^45^. Significant differential expression was defined as a log2 fold change of ≥ 2.5 (5.7x) and False Discovery Rate (FDR) value of < 0.05. SNP and INDEL identification was performed using VarScan (Version 2.3.9)^46^.

Official gene names were obtained using the HUGO Genome Nomenclature Committee multi-symbol checker accessed at: https://www.genenames.org/cgi-bin/symbol_checker. Lists of gene names were analyzed using the gene annotation tool included in the DAVID Bioinformatics Resources 6.8 accessed at https://david.ncifcrf.gov/.^15^ KEGG pathways identified from this analysis with a fold enrichment of at least 2.5 were considered significant. Genes were also analyzed using the Panther Overrepresentation test^20^ accessed at http://www.geneontology.org/. A false discovery rate (FDR) of < 0.05, using the Fisher's Exact with FDR multiple test correction, was considered significant.

### 5-azacytidine treatment of KIS-1 cells

Cells were centrifuged at 700 g for 3 min and resuspended at 1 × 10^6^ cells/ml in media with 2 µM 5-azacytidine (Sigma) or vehicle (media) for 24 h. For 48 h treatments, cell were centrifuged at 24 h and resuspended in the same volume of media with or without 2 µM 5-azacytidine.

### siRNA knockdown in KIS-1, REH and GM12878 cells

5 × 10^6^ KIS-1, KIS-1 + EBF1, GM12878 and REH cells were isolated in an actively growing state, washed with PBS and transfected with 200 nM siGENOME SMARTpool siRNAs (Dharmacon, GE Healthcare) targeting *PAX5* (M-012241-00), *KMT2A* (M-009914-01) or 200 nM non-targeting control siRNA #2 (D-001210-02). Transfection was by electroporation using the Amaxa Nucleofector device (Lonza), Kit V and the M-013 program. Transfected cells were grown in 2 mL media and samples were taken 48 h post-transfection. For combined siRNA silencing and doxycycline induction, cells were transfected with siRNA for 24 h and then doxycycline was added for a further 24 h.

### RNA preparation and RT-qPCR

RNA was prepared as described above and quantified using a Nanodrop 1000 spectrophotometer (Thermo Fisher Scientific) and then treated with Turbo DNase (Thermo Fisher Scientific). DNase-treated RNA (maximum of 5 μg) was used as template in reverse transcription reactions using oligo-dT oligonucleotide primers and SuperScript III Reverse Transcriptase (Invitrogen) following the manufacturer’s protocol. qPCR was performed using BR SYBR Green SuperMix (Quanta Biosciences) on Mastercycler ep Realplex^2^ or Realplex^4^ gradient thermocycler (Eppendorf). Mean fold changes were calculated using the 2^−ΔΔCt^ method and reflect amplification of target transcripts versus HPRT1 (which had very low variation between treatment groups) in the experimental versus the control sample. Experiments involving RT-qPCR were performed in biological triplicate (cell lines grown independently), with each data point of each replicate measured in technical triplicate. The standard errors of the mean calculations used are described in the figure legends. Figures were generated with Prism 6 (GraphPad).

Primers used for qPCR:

HPRT1 5ʹ-TGACACTGGCAAAACAATGCA-3ʹ, 5ʹ-GGTCTTTTTCACCAGCAAGCT-3ʹ.

CD19 5ʹ-ACCTGACCATGTCATTCCACCT-3ʹ, 5ʹ-AGAAGATCAGATAAGCCAAAGTCACA-3ʹ.

CD79B 5ʹ-TTGCTGCTGCTGCTCTCA-3ʹ, 5ʹ-CGCGAACAAGCACTACCTTT-3ʹ.

PAX5 5ʹ-GCGCAAGAGAGACGAAGGT-3ʹ, 5ʹ-CTGCTGCTGTGTGAACAAGTC-3ʹ.

PRDM1 5ʹ-ACGTGTGGGTACGACCTTG-3ʹ, 5ʹ-CTGCCAATCCCTGAAACCT-3ʹ.

XBP-1 5ʹ-CCGCAGCACTCAGACTACG-3ʹ, 5ʹ-TGCCCAACAGGATATCAGACT-3ʹ.

ALDH1A1 5ʹ-CCAAAGACATTGATAAAGCCATAA-3ʹ, 5ʹ-CACGCCATAGCAATTCACC-3ʹ.

TNFRSF8 5ʹ-GCTGTCAGGAGGTGCTGTTAC-3ʹ, 5ʹ-GTAGGCCTCTGTGGGCACT-3ʹ.

KMT2A 5ʹ-CCAGCCATTTGCTACGCTAC-3ʹ, 5ʹ-GGAGCTGCGGGAAGGTAT-3ʹ.

STRA6 5ʹ-GGCTGCCTACCCTTTCATCT-3ʹ, 5ʹ-CTGGGACGACATTCTCTGG-3ʹ.

S100A14 5ʹ-CTTCTGAGCTACGGGACCTG-3ʹ, 5ʹ-TTCTCTTCCAGGCCACAGTT-3ʹ.

S100A16 5ʹ-TCTTCTCCAGGGACCAGAAA-3ʹ, 5ʹ-TTCCACCAGGACAATGACTG-3ʹ.

GNA15 5ʹ-ACGTGATCGCCCTCATCTAC-3ʹ, 5ʹ-GCTCTCCTTCATGCGGTTC-3ʹ.

CD82 5ʹ-AAAGCAGAACCCGCAGAGT-3ʹ, 5ʹ-CCAGTGCAGCTGGTCACA-3ʹ.

CD79A 5ʹ-ATATGGAGCATTACCGGATCA-3ʹ, 5ʹ-GGGCTCTGTGGAGTGTTTGT-3ʹ.

### Co-immunoprecipitation

2 × 10^8^ suspension cells were harvested for nuclear purification. Cells were washed with PBS and pelleted. Nuclei were isolated and nuclear extracts obtained following the protocol of McManus et al.^7^. Briefly, cells were lysed in hypotonic lysis buffer, nuclei were recovered by centrifugation and then lysed in the presence of Benzonase nuclease (Sigma). Insoluble material following lysis was removed by centrifugation.

Nuclear protein extracts were diluted to 1 μg/μL with nuclear lysis buffer. 5 μg of the appropriate antibody (sc-1974 (C20) for PAX5 or normal goat IgGs, both from Santa Cruz Biotechnology) was added per mg of nuclear protein and incubated overnight with end-over-end mixing at 4 °C. Pre-washed Dynabeads (Life Technologies) were added and then incubated at 4 °C with end-over-end mixing for at least 1.5 h. Unbound protein was removed by magnetic sorting and beads were washed up to 5 times with co-IP buffer. Proteins associated with beads were eluted with 2 × SDS loading dye by boiling for 5 min prior to loading on polyacrylamide gels. Co-immunoprecipitations were performed in biological duplicate; representative data are shown.

### Mass spectrometry and proteomics analysis

Protein material from co-immunoprecipitation experiments was clarified (cleared of particles) by centrifugation at 2200 g. Soluble protein in the supernatant was diluted in 2 × gel loading buffer [4% w/v SDS, 100 mM TrisHCl (pH6.8), 0.2% w/v bromophenol blue, 200 mM dithiothreitol (DTT)] for separation using sodium dodecyl sulfate polyacrylamide gel electrophoresis (SDS-PAGE). Pre-cast 8% polyacrylamide gels were purchased from BioRad. Gels were fixed with 50% methanol containing 5% acetic acid for one hour, stained with EZ-Blue Gel Staining reagent (Sigma-Aldrich) for an additional hour and then de-stained in deionized water overnight. Each gel lane was excised into twelve equal bands approximately 5 mm in height. All bands were treated with 10 mM DTT for reduction of disulfide bonds followed by irreversible alkylation of all cysteine residues with 25 mM iodoacetic acid. Enzymatic protein digestion was carried out by adding 50 µL of 20 ng/µL sequencing-grade trypsin (Promega) to each band followed by incubation at 37 °C for 16 h. Upon completion of the digestion, the supernatant was removed from the gel bands and saved in a 1.5 mL Eppendorf tube. The remaining gel-bound peptides were extracted three times with 50 µL aliquots of 50% acetonitrile and 5% acetic acid. Each extract was pooled into the original 1.5 mL tube and the entire sample was concentrated by vacuum centrifugation. Peptides in the concentrate were loaded onto C18 mini-spin cartridges for clean-up by solid phase extraction. Upon completion of the equilibration, sample loading and wash steps, the peptides were eluted with 70% acetonitrile and re-concentrated by vacuum centrifugation. Samples were then diluted to 50 µL in 1% aqueous acetic acid and stored at -80C awaiting analysis by nano-liquid chromatography (nanoLC-MS/MS).

Identification of tryptic peptides for bottom-up protein analysis was performed on a hybrid quadrupole-Orbitrap (Q-Exactive, Thermo-Fisher Scientific) mass spectrometer interfaced to a Proxeon Easy nanoLC II (Thermo-Fisher Scientific). 5 µL of each sample in 1% aqueous acetic acid was injected onto a narrow-bore (100 µm i.d., x 20 mm long) C18 pre-column packed with 5 µm Reprosil-Pur resin (Thermo-Fisher Scientific). Analytical chromatographic separation was achieved on an Easy C18 column with dimensions of 75 µm inner diameter and 100 mm long. Solvents A and B for the gradient LC elution profile consisted of 0.1% aqueous formic acid and 9.9/90/0.1 water/acetonitrile/formic acid, respectively. The gradient began with 5% solvent B and was increased to 35% over 60 min. After 60 min, the gradient was stepped to 90% for 10 min and re-equilibrated to 5% B for 10 min. The LC flow rate was held constant at 300nL/min throughout the separation. Both solvents were LC–MS analytical grade from VWR. The 15 µm inner diameter electrospray ionization (ESI) emitter was biased at 1.7 kV and positioned about 2 mm from the heated (250C) ion transfer capillary. The S-lens was biased at 100 V. The Orbitrap mass analyzer was calibrated in positive ion mode at 70 k resolution every three days using a commercial calibration mixture of caffeine, the peptide MRFA and Ultramark polymer as per the instrument manufacturer’s recommendation. Mass spectrometric data was acquired in “data dependent acquisition” (DDA) mode whereby a full mass spectrum from 400 to 1200 Thomsons (Th) was followed by the acquisition of fragmentation spectra of the ten most abundant precursor ions with full-scan intensities greater than a threshold of 20,000. Precursor ion spectra were collected at a resolution of 70 k (@ 200amu) and a target value of 1E6. Peptide fragmentation was performed at 27 eV within the high energy collision induced dissociation (HCD) cell. The subsequent MS/MS spectra were collected in the Orbitrap analyzer at a resolution setting of 17.5 k and a target value of 1E5. The m/z values of the selected peptide precursor ions were placed on a dynamic exclusion list for a period of 20 s to maximize the number of peptide ions targeted for fragmentation over the course of the LC run.

Raw data files from the mass spectrometer were analyzed using Proteome Discoverer 2.0 (Thermo-Fisher Scientific) employing the Sequest HT and MS Amanda searching algorithms^47,48^. The Swissprot Homo Sapiens FASTA database (July 2018, 71,478 kb) was obtained from the Uniprot.org website. Searches were performed using the following settings: maximum of 2 missed cleavages, 10 ppm precursor mass tolerance, 0.8 Da fragment mass tolerance, dynamic modifications of methionine oxidation (+ 15.99 Da) and N-terminal acetylation (+ 42.01 Da) as well as a static modification of cysteine carboxymethylation (+ 58.005 Da). Scaffold version 4.4.5 (Proteome Software Inc.) was used to validate MS/MS based peptide and protein modifications. Identifications were accepted when their probability was greater than 95% for peptides and 99% for proteins. Protein probabilities were assigned by Protein Prophet^49^ and proteins that could not be differentiated based on unique spectra were grouped to satisfy the principle of parsimony. Gene lists derived from the protein identifications were analyzed with the Panther Overrepresentation test, as described above.

Proteins were classified as having a minor increase in association with PAX5 in the presence of EBF1 when 1.5 × or more unique peptides were identified in EBF1+ versus EBF1− in each of the two biological replicate experiments.

## Data availability

RNA-seq data has been deposited in the Gene Expression Omnibus (GEO) database under the following Accession number: GSE136920. Proteomics data has been deposited in the MassIVE repository, a full member of the PRIDE repository, with the following accession number: MassIVE ID: MSV000084234.

## Supplementary Information

Supplementary Information 1.

Supplementary Information 2.
