# Supplementary material for: EBF1 drives hallmark B cell gene expression by enabling the interaction of PAX5 with the MLL H3K4 methyltransferase complex
Source: Sci Rep. 2021 Jan 15;11:1537. doi: 10.1038/s41598-021-81000-5 (PMC7810865; doi:10.1038/s41598-021-81000-5)

Title: EBF1 drives hallmark B cell gene expression by enabling the interaction of PAX5 with the MLL H3K4 methyltransferase complex

Authors: Charles E. Bullerwell, Philippe Pierre Robichaud, Pierre M.L. Deprez, Andrew P. Joy, Gabriel Wajnberg, Darwin D’Souza, Simi Chacko, Sébastien Fournier, Nicolas Crapoulet, David A. Barnett, Stephen M. Lewis, Rodney J. Ouellette

Supplementary Table Legends

Supplementary Table S1. Differential gene expression in KIS-1 versus RAJI as determined by RNA-seq

Sheet 1: all data, with genes showing a differential expression of <2.5 or >-2.5 log2 fold change indicated in red

Sheet 2: all data with pdj (FDR) ≤0.05, with genes showing a differential expression of <2.5 or >-2.5 log2 fold change indicated in red

Supplementary Table S2. SNPs and Indels in KIS-1 based on RNA-seq data

Sheet 1: SNPs

Sheet 2: Indels

Supplementary Table S3. Significantly differentially-expressed genes in the presence or absence of EBF1 expression

a KIS+empty +/-DOX

b KIS1+EBF1 –DOX vs KIS1+empty –DOX

c KIS1+EBF1 +/-DOX

Sheet 1: all data, with genes showing a differential expression of <2.5 or >-2.5 log2 fold change indicated in red

Sheet 2: all genes showing a differential expression of <2.5 or >-2.5 log2 fold change with pdj (FDR) ≤0.05

d KIS1+EBF1 +DOX vs KIS1+empty +DOX

Sheet 1: all data, with genes showing a differential expression of <2.5 or >-2.5 log2 fold change indicated in red

Sheet 2: all genes showing a differential expression of <2.5 or >-2.5 log2 fold change with pdj (FDR) ≤0.05

e Upregulated genes from KIS1+EBF1 +/-DOX and/or KIS1+EBF1 +DOX vs KIS1+empty +DOX

Supplementary Table S4. Proteins associated with PAX5 in KIS-1+EBF1+/-DOX as determined by Mass Spectrometry

Sheet 1: Unique peptide counts

Sheet 2: Combined proteins found to interact with PAX5 in both DOX+ and DOX- (at least 2 peptides identified in each replicate)

Sheet 3: Proteins with increased PAX5 binding in the presence of DOX (≥1.5-fold unique peptides in DOX+ versus DOX- and at least 2 unique peptides in each DOX+ replicate.

Sheet 4: Proteins with decreased PAX5 binding in the presence of DOX (≥1.5-fold unique peptides in DOX- versus DOX+ and at least 2 unique peptides in each DOX- replicate.

Sheet 5: PAX5-associated proteins assigned to Gene Ontology groups using the Panther overrepresentation test

Sheet 6: Proteins with increased PAX5 binding in the presence of DOX assigned to Gene Ontology groups using the Panther overrepresentation test

Supplementary Table S5. KMT2 complex components (see Rao and Dou, 2015) identified in proteomics data following immunoprecipitation of PAX5 (C20) or using non-specific, control antibodies (IgG).

Number of unique peptides identified by mass spectrometry is indicated.

		KIS-1+EBF1				NALM6				RAJI			
		+DOX	-DOX	+DOX	-DOX								
		Rep1	Rep1	Rep2	Rep2	Rep1	Rep2	Rep1	Rep2	Rep1	Rep2	Rep1	Rep2
		C20	C20	C20	C20	C20	C20	IgG	IgG	C20	C20	IgG	IgG
	PAX5	17	20	19	17	18	15	0	0	18	15	0	0
**Subunits common to KMT2 complexes**	ASH2L	5	0	1	1	0	0	0	0	2	2	0	0
	RBBP5	3	1	2	1	4	0	0	0	2	2	0	0
	WDR5	5	3	2	1	3	6	0	0	4	0	0	0
	DPY30	1	0	0	0	0	0	0	0	0	0	0	0
**Subunits unique to KMT2A/2B complex**	KMT2A	9	3	5	3	8	2	0	0	0	12	2	0
	KMT2B	0	0	0	0	0	0	0	0	0	0	3	0
	MEN1	10	6	7	7	0	0	0	0	3	3	0	0
	HCFC1 or	5	1	0	0	12	6	0	0	4	9	0	0
	HCFC2	0	0	0	0	0	0	0	0	0	0	0	0
**Subunits unique to KMT2C/2D complex**	KMT2C	0	0	0	0	0	0	0	0	0	0	2	0
	KMT2D	0	1	0	0	0	0	0	0	0	0	3	0
	PAXIP1	0	0	0	0	2	0	0	0	0	0	0	0
	PAGR1	0	0	0	0	0	0	0	0	0	0	0	0
	NCOA6	0	1	0	0	0	0	0	0	0	0	2	0
	KDM6A	0	0	0	0	0	0	0	0	0	0	0	0
**Subunits unique to KMT2F/2G complex**	KMT2F	0	0	0	0	0	0	0	0	0	0	0	0
	KMT2G	0	0	0	0	0	0	0	0	0	0	0	0
	CXXC1	0	0	0	0	6	2	0	0	0	0	0	0
	WDR82	0	0	0	0	3	0	0	0	0	0	0	0
	HCFC1	5	1	0	0	12	6	0	0	4	9	0	0

Supplementary Figures

Supplementary Figure S1. Western blot of MEN1 and KMT2A following 48h DOX induction of KIS-1+EBF1 and KIS-1+empty cells shown by Western blotting.

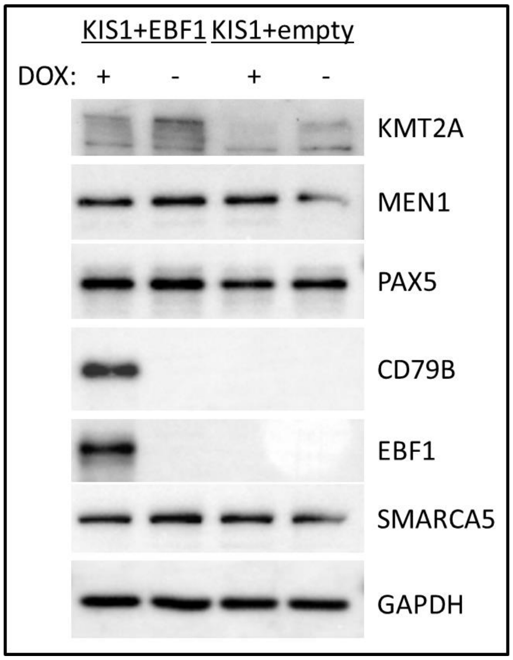

Supplementary Figure S2. Activation of CD19 (A) and CD79b (B) expression following exogenous expression of a control gene (CAT) or EBF1 in HEK293T cells shown by RT-PCR

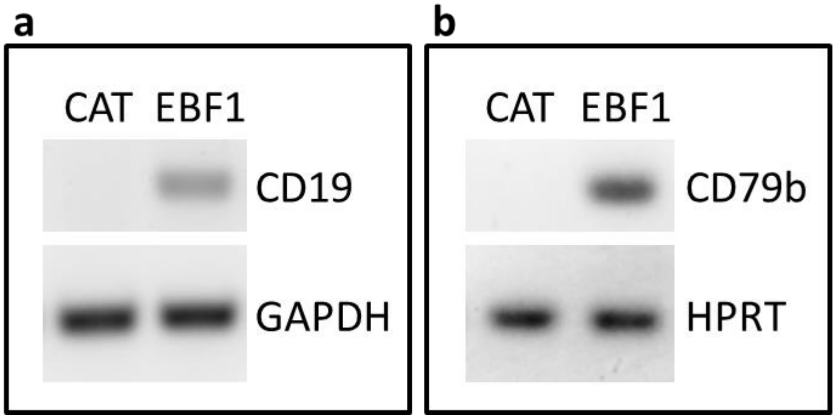

Supplementary Figure S3. Western blot of CD19 and CD79b following silencing of PAX5 (a) and EBF1 (b) in REH cells

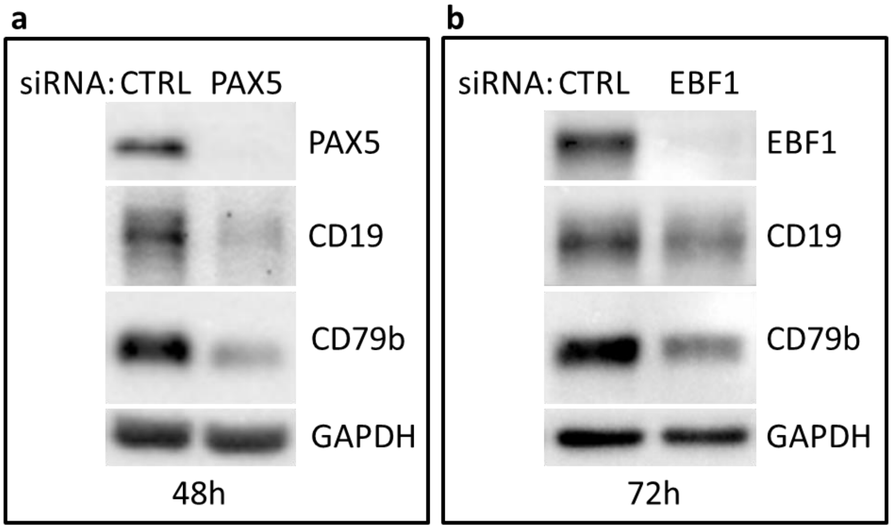

Supplementary Figure S4. Native chromatin immunoprecipitation coupled with quantitative PCR (N-ChIP-qPCR). Native chromatins have been immunoprecipitated from EBF1 inducible KIS-1 cells, incubated with or without 100 ng/ml DOX for 48h, using negative control IgG (**A**), H3K4me2 (**B** and **D**) and H3K4me3 (**C** and **E**) antibodies. Several promoter and UTR/CDS regions of CD19 and CD79B genes as well as one negative control (AM NEG) and two positive controls (cFOS and HPRT) were quantified by qPCR. Data are expressed as percentage of input (**A**, **B** and **C**) and as fold enrichment (**D** and **E**). Data are the means ± SEM of 3 independent experiments (n=3).

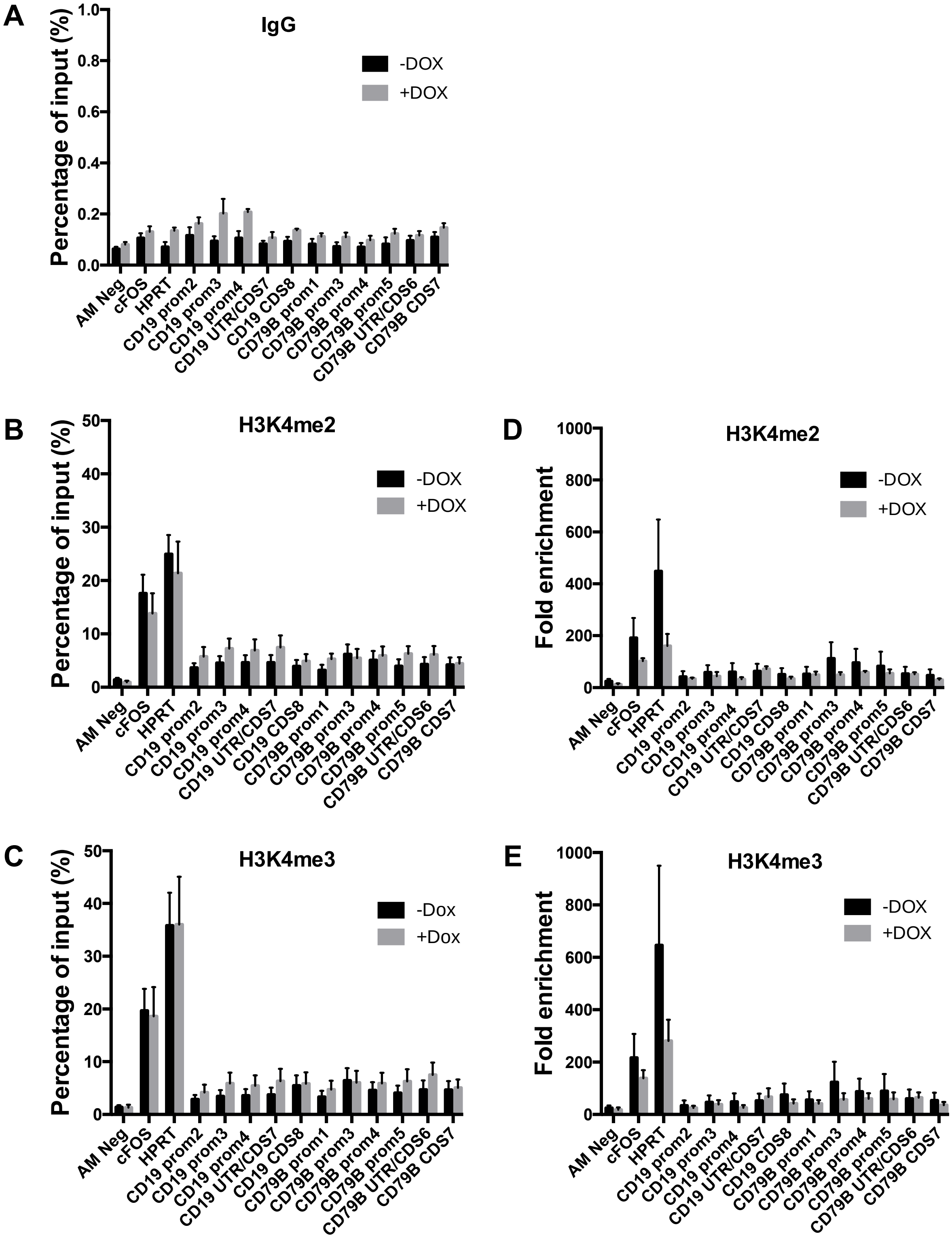

Supplementary Methods

Growth and transfection of HEK293T cells (Supplementary Figure S2).

HEK293T cells were obtained from the American Type Culture Collection (ATCC CRL-3216) and cultured in DMEM high glucose media supplemented with 10% FBS, 2mM L-glutamine and 1x Glutamax (GIBCO). Cells were trypsinized and split 1/10 with media every three days.

The EBF1 coding sequence was PCR amplified using Taq polymerase *Pfx* from plasmid MHS6278-202758239 (Dharmacon) using the primers 5'-CCCTCGTAAAGAATTATGTTTGGGATTCAGGAAAGCATCCAACG-3' and 5'-GTGTATACGGGAATTTCACATAGGAGGAACAATCATGCCAGATATCG-3' and CloneAmp HiFi PCR Premix (Clontech). Primers for PCR amplification were designed to add the flanking attB sequences necessary for subsequent Gateway® cloning. Amplicons were cloned into pDONR 221 (Invitrogen) and then transferred into the expression vector pcDNA3.2 V5 DESTplasmid (Invitrogen) without the STOP codon in order to fuse the ORF to a V5 peptide tag. pcDNA3.2/V5/GW-CAT (Invitrogen) was used as a negative control. All cloned DNA fragments were verified by sequencing.

HEK293T cells were transfected at approximately 75% confluence. Cells grown in 6-well plates were washed with PBS and then transfected with 4μg plasmid and 10μL Lipofectamine 2000 (Invitrogen) in a final volume of 2.5mL of media. Cells were maintained in an actively growing state by splitting transfections by half at 24h post-transfection. Cells were harvested at 48h post-transfection.

Native chromatin immunoprecipitation coupled with quantitative PCR (N-ChIP-qPCR) (Supplementary Figure S4).

Native chromatin immunoprecipitation were performed using the Chromatrap® Native ChIP pro G kit (#500238) following the manufacturer protocol. Briefly, native chromatins were prepared from $15x10^6$ of stably transduced KIS-1 cells with lentiviral EBF1 inducible vector treated with 100ng/ml doxycycline (DOX+) or vehicle (water; DOX-) for 48h. Nanodrop quantified chromatins were enzymatically sheared using 1U of shearing cocktail per 5 ug of chromatin as recommended by the manufacturer protocol. The chromatin shearing efficiencies were verified using the Advanced Analytical 5200 fragment analyzer (Agilent) and a ratio of 2 ug of antibody per 5 ug of chromatin has been used to perform the immunoprecipitation as recommended. The control antibody IgG (#2729S) was from cell signaling and the anti-H3K4me2 (#ab7766) and the anti-H3K4me3 (#ab8580) antibodies were from abcam. Immunoprecipited DNA were extracted and several regions of CD19 and CD79B genes promoters/UTR/CDS as well as one negative control (AM NEG) and two positive controls (cFOS and HPRT) were quantified by qPCR using the 2x BR SYBR® Green SuperMix (Quanta Biosciences) on a CFX connect qPCR instrument (Bio-Rad). Data have been express as percentage of input and fold enrichment.

Primers used for qPCR:

AM NEG, Active Motif ChIP Human Negative Control Primers Set 1 (#71001)

cFOS 5’- TTAGGACATCTGCGTCAGCAGGTT-3’, 5’-TCTCGTGAGCATTTCGCAGTTCCT-3’

HPRT 5’- GTTGGGAGGGAAAGGGGCTTC-3’, 5’- ACGCCGGCGCCTACCAGTT-3’

CD19 prom2 5’-GAGAAGGAGTCTATGTGCCCAGCA-3’, 5’-CTGCACAAGAATGTGAGCCCCTTG-3’

CD19 prom3 5’- CAAGGGGCTCACATTCTTGTGCAG-3’, 5’-CTGCCACGCTGTTTTATTTTCATCCCA-3’

CD19 prom4 5’- TGGGATGAAAATAAAACAGCGTGGCAG-3’, 5’-GAGGAAGGCGGTGGTCACG-3’

CD19 UTR/CDS7 5’- CCTCTTCTTCCTCCTCTTCCTCACC-3’, 5’-CCCTTCCCTTTCTGCCCTTTGG-3’

CD19 CDS8 5’- CCAAAGGGCAGAAAGGGAAGGG-3’, 5’-TAAGAAAATGGAGGCTCAGAGAGGGTAAGT-3’

CD79B prom1 5’- GCTCACGGCCCAGGAATAGAG-3’, 5’-ACCGGTGGTCATCCCCTGG-3’

CD79B prom3 5’- CCAGAGGCATCCACAGAGGAC-3’, 5’-CCTGGGGGAGGGCAGGCTT-3’

CD79B prom4 5’- AAGCCTGCCCTCCCCCAGG-3’, 5’- CCGCCTCTTCCTCACCAGG-3’

CD79B prom5 5’- CCTGGTGAGGAAGAGGCGG-3’, 5’-ACCCCAAACCCGTGACAACG-3’

CD79B UTR/CDS6 5’- CGTTGTCACGGGTTTGGGGT-3’, 5’-CAGCAGCAGCAACGCCACCA-3’

CD79B CDS7 5’- TGGTGGCGTTGCTGCTGCTG-3’, 5’- GAGGCAGAGCCGCAGGGC-3’

Expanded Data Supplementary File With Full Western Blots

MW marker used for all Western Blots:

Bio-Rad Precision Plus Protein™ Dual Color Standards

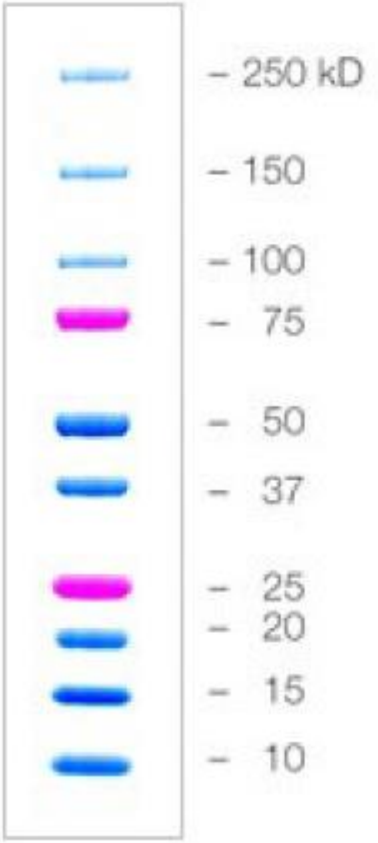

Figure 1A: Full-membrane Western blot data (* indicates remaining signal from a previous blot on the membrane)

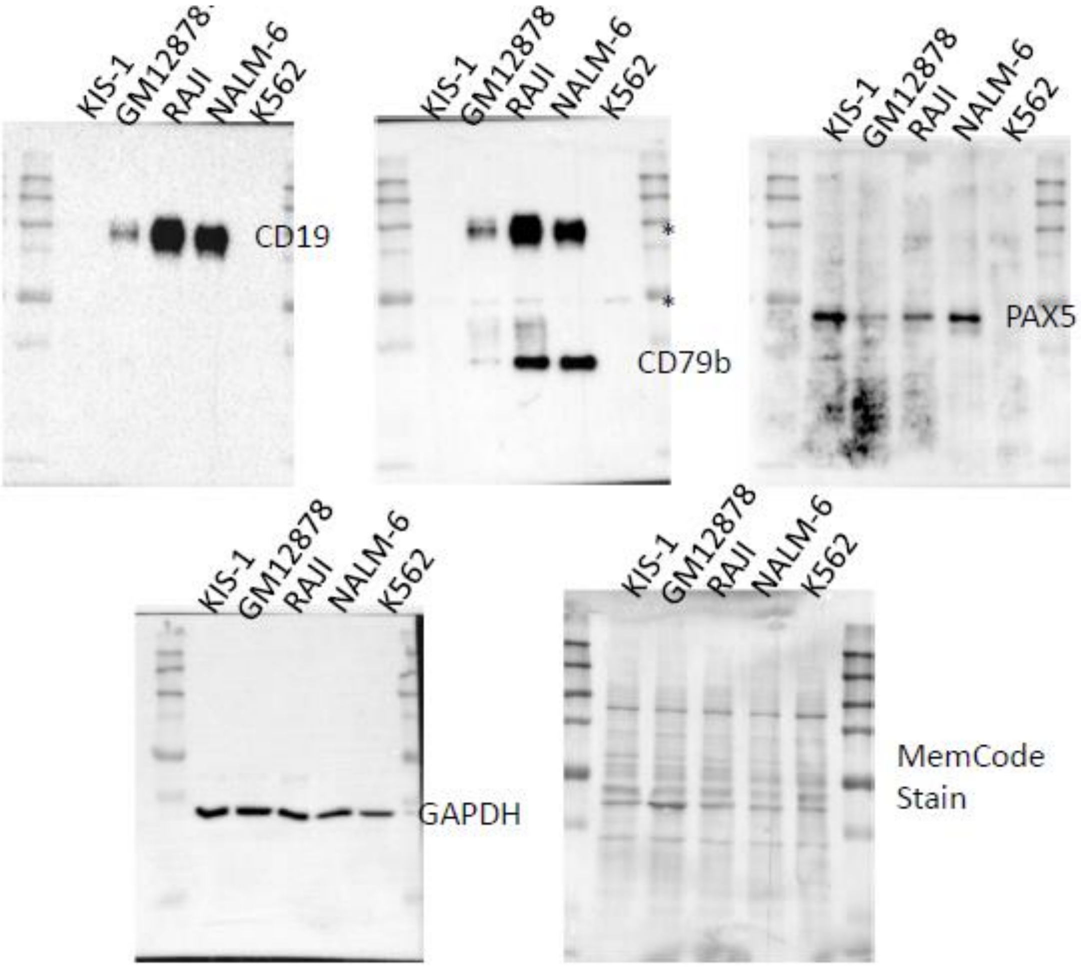

Figure 5C: Full-membrane Western blot data. (* indicates remaining signal from a previous blot on the membrane)

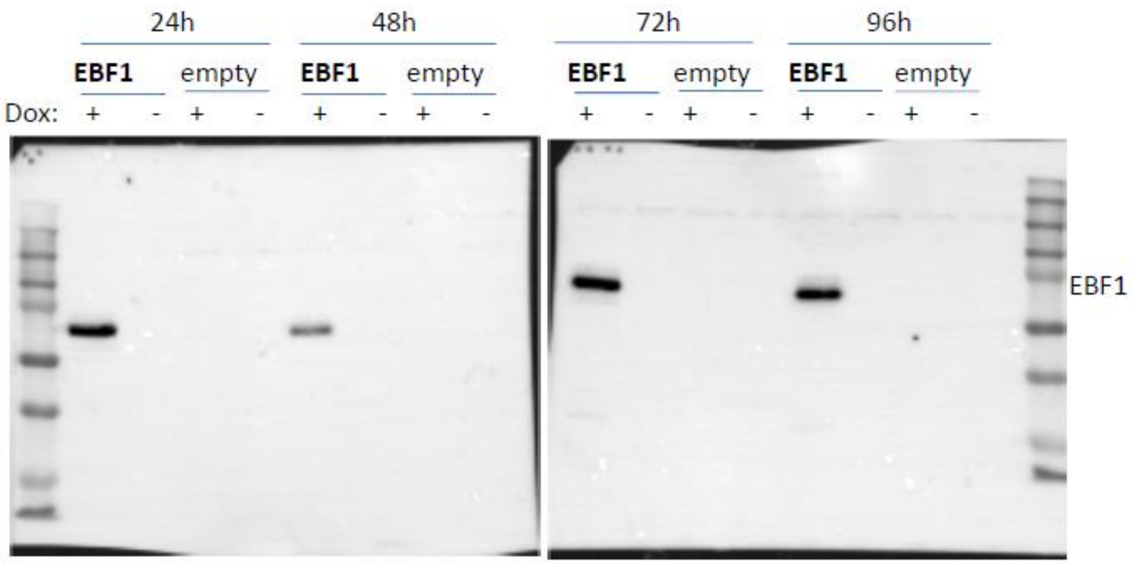

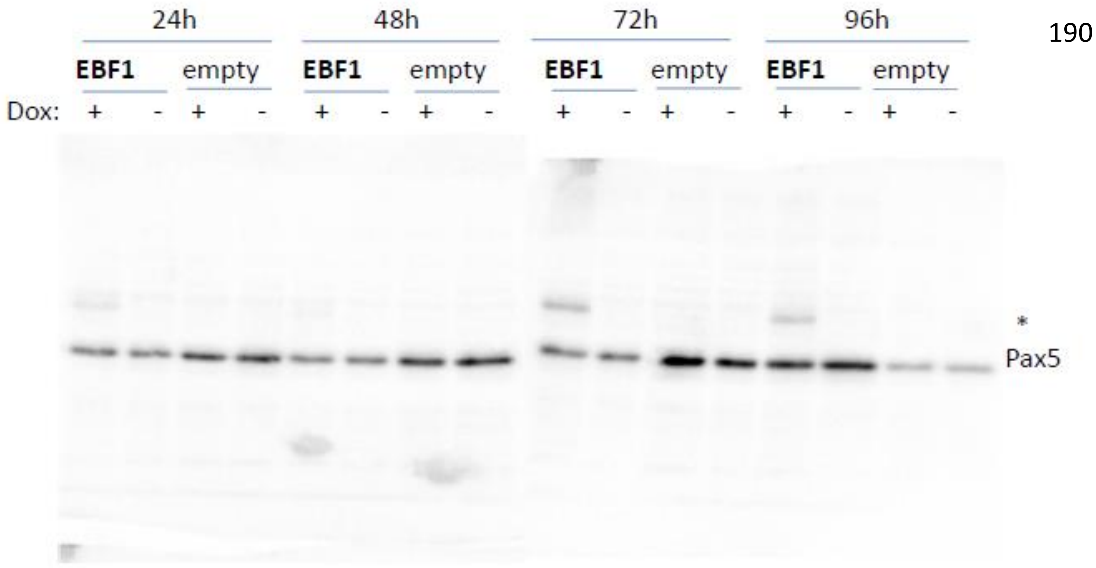

Figure 5C: Full-membrane Western blot data (* indicates remaining signal from a previous blot on the membrane)

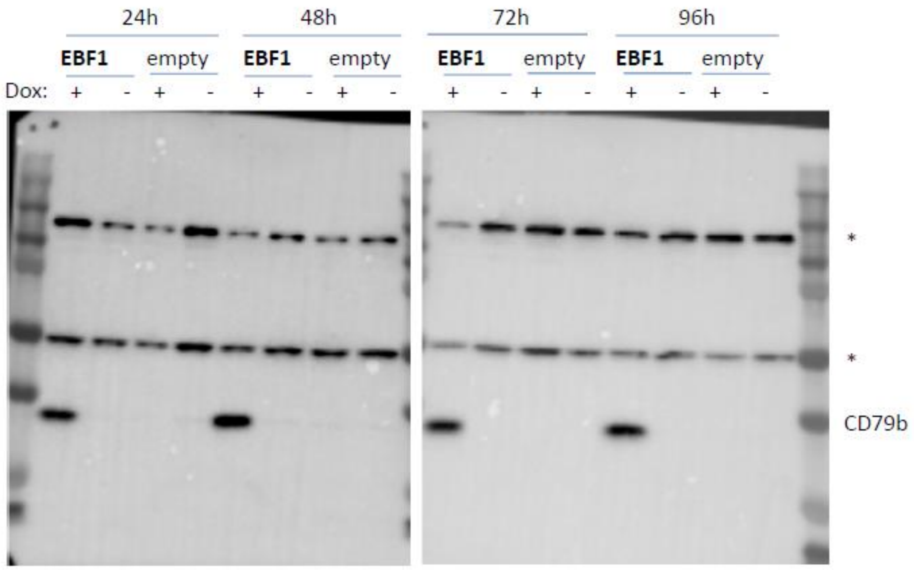

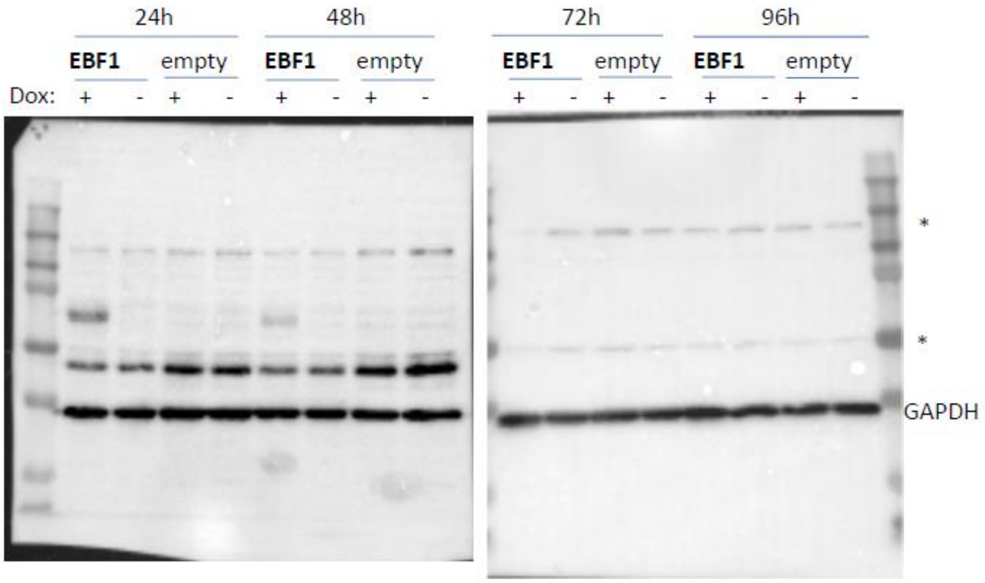

Figure 6A: Full-membrane Western blot data (* indicates remaining signal from a previous blot on the membrane)

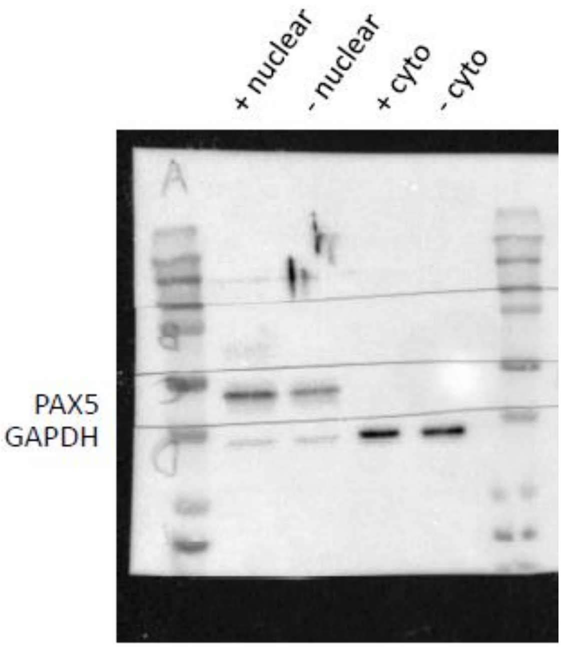

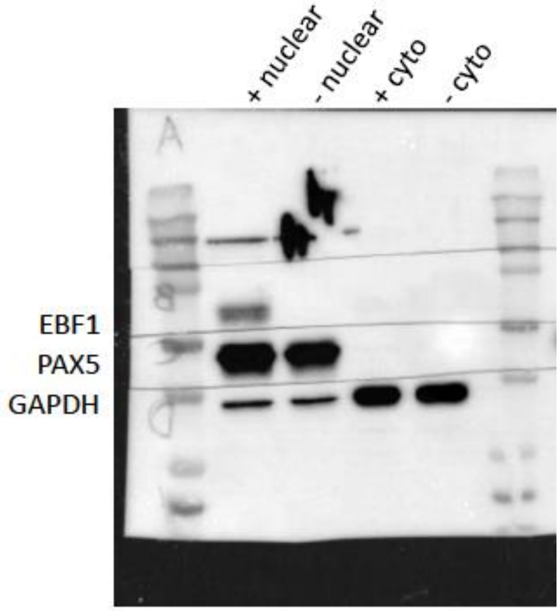

Original blots were cut for hybridization with different antibodies and then re-assembled

Figure 6B: Coomassie staining of the second biological replicate PAX5 immunoprecipitations used for proteomic identification of PAX5-binding proteins.

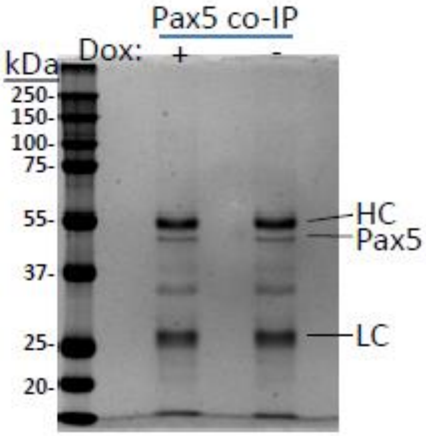

Figure 6C: Full membranes

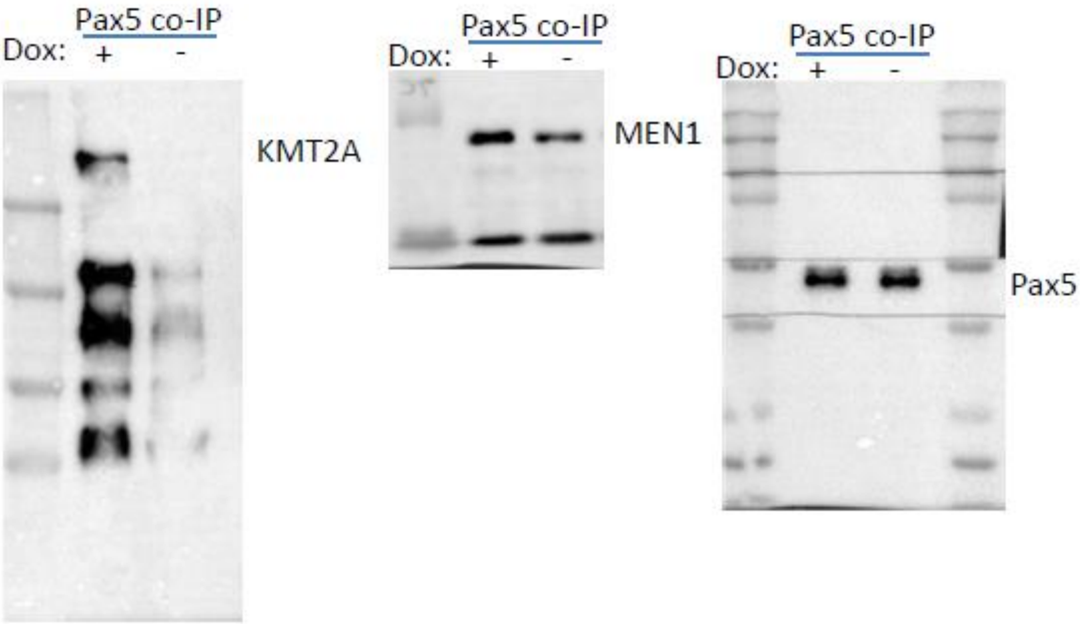

Original blots were cut for hybridization with different antibodies and then re-assembled

Figure 6D: Full membranes

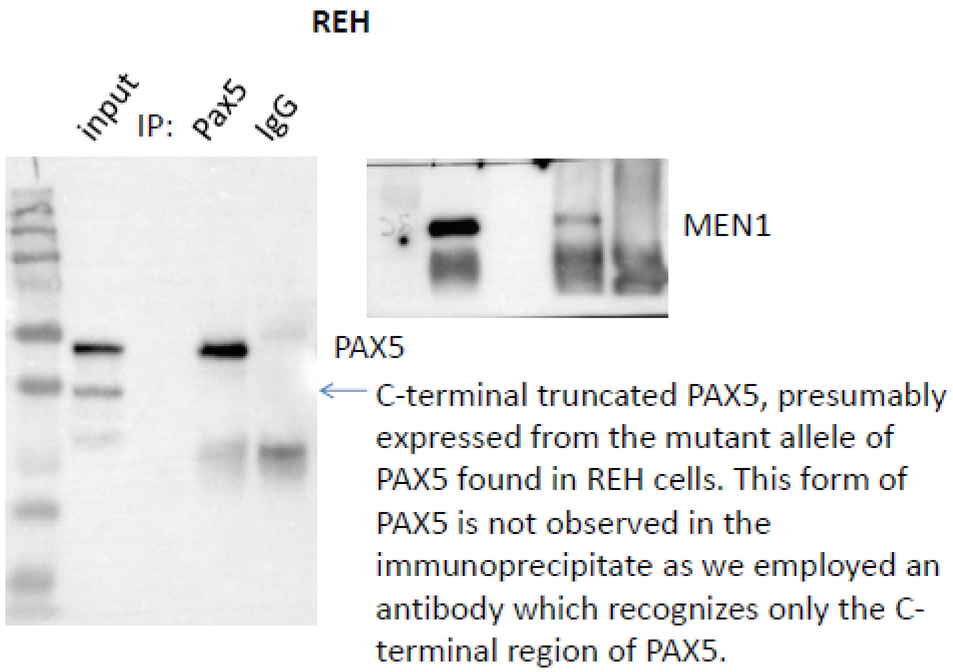

Figure 6D: Full membranes

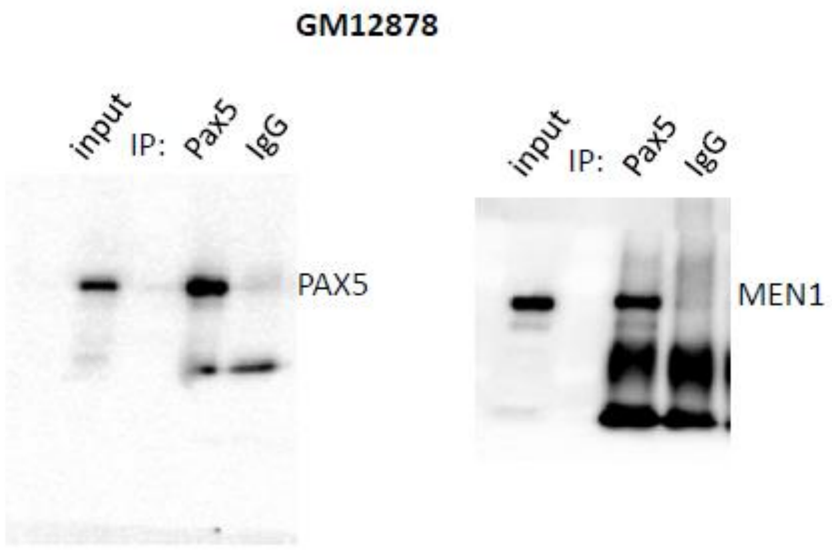

Supplement: Supplementary file 2 — Supplementary Information 2. [file 41598_2021_81000_MOESM2_ESM.pdf]
